# An intra-patient contemporaneous comparison of ^18^F-piflufolastat and ^18^F-flotufolastat urinary radioactivity and pelvic region detection rates in men with low PSA biochemical recurrence of prostate cancer after radical prostatectomy

**DOI:** 10.1007/s00259-025-07732-y

**Published:** 2026-02-23

**Authors:** Luke T. Nordquist, Jack R. Andrews, Phillip H. Kuo, Benjamin A. Gartrell, David Josephson, Andrei S. Purysko, Daniel R. Saltzstein, Ram A. Pathak, Neal Shore, James Sykes, Ross Penny, Phillip Davis, Brian T. Helfand

**Affiliations:** 1 XCancer Corporation, Omaha, NE USA; 2 Mayo Clinic, Phoenix, AZ USA; 3 City of Hope National Medical Center, Duarte, CA USA; 4 Montefiore Einstein Comprehensive Cancer Center, Bronx, NY USA; 5 Tower Urology, Los Angeles, CA USA; 6 Cleveland Clinic, Cleveland, OH USA; 7 Urology San Antonio, San Antonio, TX USA; 8 Mayo Clinic, Jacksonville, FL USA; 9 START Carolinas, Myrtle Beach, SC USA; 10 Blue Earth Diagnostics Ltd, Oxford, UK; 11 Blue Earth Diagnostics, Inc., Needham, MA USA; 12 Endeavor Health, 2650 Ridge Avenue, Evanston, IL 60201 USA

**Keywords:** ^18^F-Piflufolastat, ^18^F-Flotufolastat, PSMA-PET, Urinary radioactivity, Intra-patient comparison

## Abstract

**Purpose:**

This prospective, multicentre, intra-patient comparator study assessed urinary radioactivity, and patient-level and region-level detection rates (DR) with PSMA-PET radiopharmaceuticals, ^18^F-piflufolastat (^18^F-DCFPyL) and ^18^F-flotufolastat (^18^F-rhPSMA-7.3) in patients with biochemical recurrence (BCR) of prostate cancer to evaluate the hypothesis that lower urinary radioactivity is observed with ^18^F-flotufolastat.

**Methods:**

Patients with low PSA (≤0.5 ng/mL) BCR ≥6 months post-prostatectomy with undetectable PSA post-surgery, scheduled for standard-of-care ^18^F-piflufolastat PSMA-PET were enrolled. Patients underwent PET/CT 60 minutes post-^18^F-piflufolastat (9 mCi) administration, and a second PET/CT on the same scanner 1–10 days later, 60 minutes post-^18^F-flotufolastat (8 mCi) administration. The primary endpoint was the difference in urinary radioactivity (SUV_mean_) between the radiopharmaceuticals. Secondary endpoints included patient-level and region-level DR for each radiopharmaceutical, assessed by two blinded readers (a third resolved disagreements, allowing majority reads).

**Results:**

Fifty-five evaluable patients (mean PSA, 0.28 ng/mL) were enrolled. Median bladder SUV_mean_ was significantly higher with ^18^F-piflufolastat (29.0; interquartile range, 18.9–40.8) than ^18^F-flotufolastat (10.9; interquartile range, 6.0–18.5; *p* < 0.001 [Wilcoxon signed-rank test]). Majority read patient-level DR were 27.3% (15/55) for ^18^F-piflufolastat and 45.5% (25/55) for ^18^F-flotufolastat. Region-level DR for ^18^F-piflufolastat and ^18^F-flotufolastat, were 10.9% (6/55) and 18.2% (10/55) in the prostate bed, 14.5% (8/55) and 16.4% (9/55) in pelvic lymph nodes, and 7.3% (4/55) and 21.8% (12/55) in extra-pelvic sites. Among patients with PSA ≤0.2 ng/mL, 38.1% (8/21) and 52.4% (11/21) had positive ^18^F-piflufolastat and ^18^F-flotufolastat scans, respectively.

**Conclusions:**

This intra-patient comparator shows ^18^F-flotufolastat has significantly lower urinary radioactivity than ^18^F-piflufolastat, which may help to optimise image assessment in regions close to the urinary tract.

**Clinical trial registration:**

Trial registration, clinicaltrials.gov: NCT06604442. Registered September 2024.

**Supplementary Information:**

The online version contains supplementary material available at 10.1007/s00259-025-07732-y.

## Introduction

It is estimated that 20–40% of patients with prostate cancer who have undergone radical prostatectomy will experience biochemical recurrence (BCR) within 10 years of treatment [1]. BCR after surgery is defined as a detectable prostate-specific antigen (PSA) level ≥ 0.2 ng/mL with a second confirmatory level > 0.2 ng/mL in a patient who had undetectable (or nadir <0.2 ng/mL) PSA following surgery [2]. Current BCR definitions were derived based on the sensitivity of conventional imaging modalities. However, positron emission tomography (PET)-based radiopharmaceuticals, including those targeting prostate-specific membrane antigen (PSMA), are more sensitive and can identify subcentimetre metastases that are often missed by conventional imaging, facilitating earlier detection of recurrent disease [3–5].

To date, two ^18^F-labeled PSMA-PET radiopharmaceuticals (^18^F-piflufolastat [^18^F-DCFPyL] and ^18^F-flotufolastat [^18^F-rhPSMA-7.3]) and one ^68^Ga-labeled PSMA-PET radiopharmaceutical (^68^Ga-PSMA-11) have been approved by the United States Food and Drug Administration for imaging in men with PSMA-positive prostate cancer who have suspected metastases and are candidates for initial definitive therapy, or who have suspected disease recurrence based on elevated PSA levels [6–8].

^18^F-Piflufolastat, ^18^F-flotufolastat, and ^68^Ga-PSMA-11 are primarily renally cleared [9, 10]. High urinary radioactivity in the ureters, and/or bladder can obscure or appear indistinguishable from disease in the pelvic or retroperitoneal lymph nodes, prostate/prostate bed, and surrounding areas [11–13], which may impact image interpretation. Several quantitative studies have measured urinary radioactivity of ^68^Ga-PSMA-11 and ^18^F-piflufolastat using standardised uptake value (SUV) metrics in the bladders of patients with prostate cancer. Donswijk et al. measured maximum standardised uptake value (SUV_max_) in a volume of interest (VOI) manually placed over the region of highest activity in the bladder of 202 patients with intermediate or high-risk primary or BCR of prostate cancer [10]. Their data show a median SUV_max_ of 43.5 for ^68^Ga-PSMA-11 and 61.7 for ^18^F-piflufolastat [10]. Giesel et al. measured a median bladder SUV_max_ in a 2 cm sphere inside the parenchyma of the bladder of 79.3 for ^18^F-piflufolastat in a pilot study of 12 patients with newly diagnosed prostate cancer [14], and Uprimny et al. measured multiple SUV parameters in VOIs drawn automatically with a manually adapted isocontour threshold centred on the bladder of patients with prostate cancer, and showed the ^68^Ga-PSMA-11 median SUV_max_ to be 68.4 and mean SUV (SUV_mean_) to be 45.8 (*n* = 50) [15].

Nonclinical and early clinical data suggest that ^18^F-flotufolastat yields high uptake in the tumour while minimising urinary radioactivity at the time of imaging [9, 16], which may facilitate improved image interpretation and detection rates compared with other PSMA-targeted radiopharmaceuticals. These data are supported by a post-hoc analysis of PET scans from 718 patients with newly diagnosed or suspected BCR of prostate cancer who were enrolled in either the Phase 3 LIGHTHOUSE [17] or SPOTLIGHT [18] study [19]. Kuo et al. reported relatively low urinary radioactivity for ^18^F-flotufolastat (median SUV_max_, 17.1; median SUV_mean_, 12.5) determined in a region of interest placed over the maximum radioactive diameter within the bladder [19]. Furthermore, scans from 96% (682/712) of patients were scored as either 0 (no urinary activity in the bladder) or 1 (possible to distinguish disease from urinary activity) by majority read, which indicated that disease assessment with ^18^F-flotufolastat was minimally impacted by the relatively low levels of urinary radioactivity [19]. A subsequent post-hoc analysis of LIGHTHOUSE and SPOTLIGHT data by Penny et al. quantitatively assessed SUV in a VOI over the entire radioactive bladder volume and also showed low urinary radioactivity of ^18^F-flotufolastat (median SUV_mean_, 10.6; median peak SUV [SUV_peak_], 16.0) [20].

When considered together, these studies suggest that ^18^F-flotufolastat PET has lower urinary radioactivity than other PSMA-targeted radiopharmaceuticals, which may improve image interpretation of lesions in the prostate/prostate bed and surrounding areas. Improved image interpretation owing to the lower urinary radioactivity may also contribute to the high (≥95% in newly diagnosed patients and ≥ 75% in the recurrent population) inter-reader agreement observed with ^18^F-flotufolastat [21], and to the differences in overall detection rates reported from Phase 3 studies of patients with suspected BCR of prostate cancer. For instance, the overall detection rate of 83% reported for ^18^F-flotufolastat in the SPOTLIGHT study [18], compares favourably with rates reported from Phase 3 studies of ^18^F-piflufolastat (59–66%) [22] and ^68^Ga-PSMA-11 (75%) [23]. Observed differences in detection rates are particularly notable among the data reported from patients with low PSA levels (<0.5 ng/mL), where ^18^F-flotufolastat was shown to have a detection rate of 64% [18], compared with reported rates of 36% for ^18^F-piflufolastat [22] and 38% for ^68^Ga-PSMA-11 [23].

While such findings may suggest that ^18^F-flotufolastat has a more optimal diagnostic performance than other PSMA-targeted radiopharmaceuticals, head-to-head, intra-patient comparisons are needed to reduce the impact of inter-patient variability on findings and to confirm any differences in urinary radioactivity and detection rates under identical acquisition conditions. Here, we present the first intra-patient comparison of ^18^F-piflufolastat and ^18^F-flotufolastat to evaluate the urinary radioactivity and detection rate of both radiopharmaceuticals in men with low PSA BCR of prostate cancer after radical prostatectomy.

## Methods

This was a multicentre, prospective, intra-patient comparator study of urinary radioactivity in patients with low PSA BCR of prostate cancer following radical prostatectomy (NCT06604442). The study was conducted in accordance with the Declaration of Helsinki and the International Council on Harmonisation Guidelines for Good Clinical Practice. The study protocol (Supplementary Appendix) was approved by each study site’s Institutional Review Board/Independent Ethics Committee prior to initiation. All patients provided written informed consent prior to study participation.

### Patients

Men (≥18 years old) were eligible if they had a history of localised adenocarcinoma of the prostate for which they had undergone curative-intent radical prostatectomy with undetectable PSA post-surgery ≥6 months previously and were suspected to have BCR owing to a rising PSA level that was ≤0.5 ng/mL. All eligible patients were scheduled to undergo ^18^F-piflufolastat PET as part of their routine clinical care. Any patient who had already undergone ^18^F-piflufolastat PET for BCR in the 8 weeks before screening, who had undergone salvage therapy for the current episode of BCR, who had undergone cystectomy, or who had renal failure or other conditions affecting urinary output (as determined by the Investigator) was excluded. Full inclusion and exclusion criteria are provided in the study protocol (Supplementary Appendix).

### Imaging procedures

**Fig. 1:**
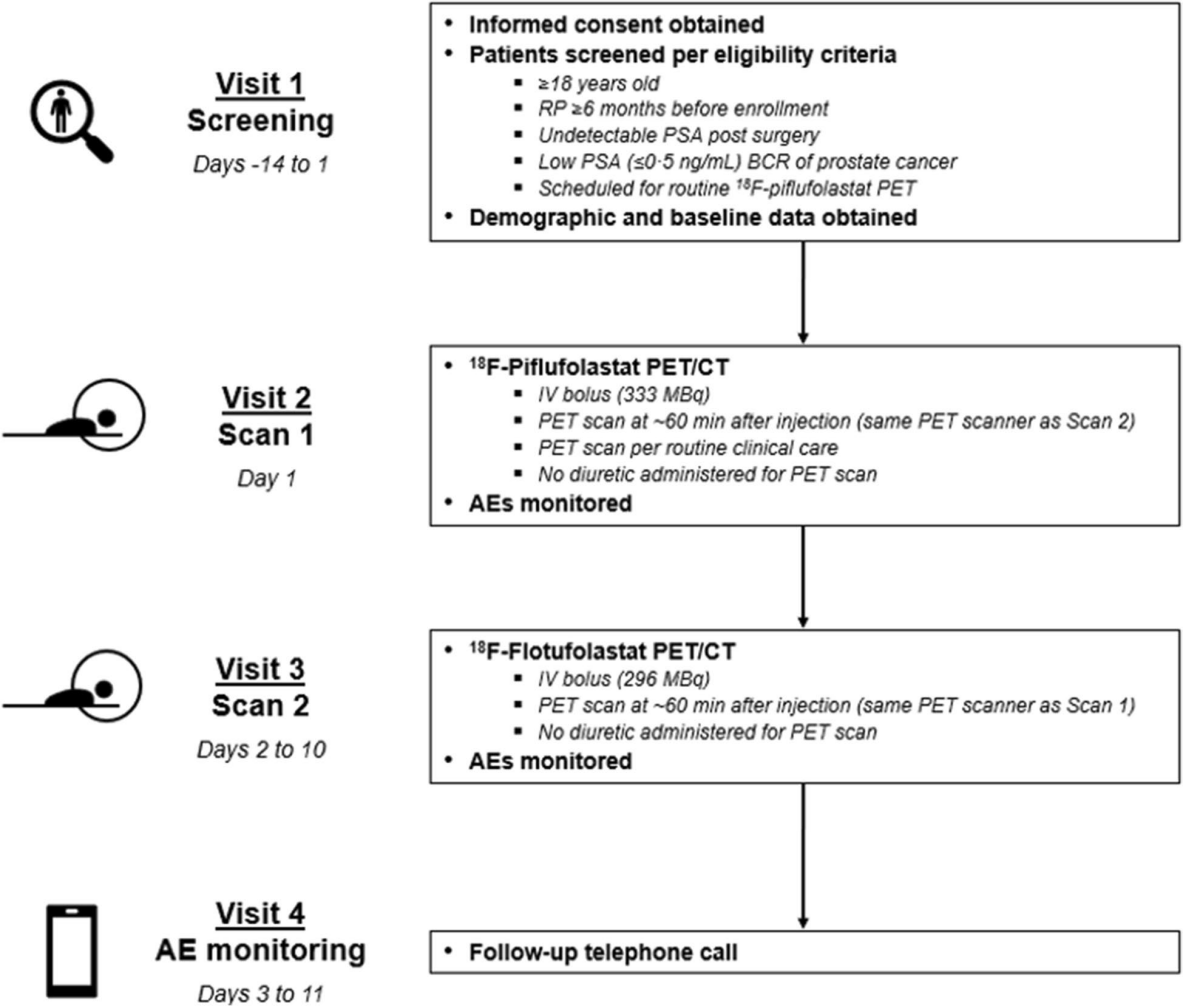
Study design and imaging workflow for intra-patient comparison of ^18^F-piflufolastat and ^18^F-flotufolastat. For each patient, the ^18^F-piflufolastat PET/CT and ^18^F-flotufolastat PET/CT were performed on the same scanner. A non-contrast CT scan was utilised for attenuation correction and anatomic localisation only. All PET scans were sent to the imaging core lab for subsequent blinded review. ^18^F=Fluorine-18. AE = adverse event. BCR = biochemical recurrence. CT = computed tomography. IV = intravenous. PET = positron emission tomography. PSA = prostate-specific antigen. RP = radical prostatectomy

All scanners used in this study were qualified by an independent imaging core laboratory, using a Jaszczak or NEMA IEC body phantom scan acquired and reconstructed using the investigating site’s typical ^18^F clinical parameters. The time per bed position used in the study was two or three minutes (or equivalent for continuous couch motion systems). ^18^F-Flotufolastat PET/CT scans were performed with equivalent scan parameters and using the same scanner as the ^18^F-piflufolastat PET/CT. Imaging procedures are outlined in Fig. 1.

For both PET acquisitions, patients were encouraged to be well-hydrated prior to the scan and were instructed to void immediately before being positioned in the PET/computed tomography (CT) scanner for image acquisition. Scans were conducted in the caudal–cranial direction beginning at mid-thigh. No diuretic was administered for scanning purposes, and the CT acquisition was performed for anatomic localisation and attenuation correction only.

#### ^18^F-Piflufolastat PET

Eligible patients underwent routine ^18^F-piflufolastat PET/CT within 0–14 days of screening. ^18^F-Piflufolastat (9 mCi [333 MBq], with an acceptable range of 8–10 mCi [296–370 MBq]) was administered as an intravenous bolus per standard institutional practice and approved US prescribing information, and scans started approximately 60 minutes post-injection [7].

#### ^18^F-Flotufolastat PET

^18^F-Flotufolastat PET/CT was scheduled to take place at least 24 hours, and no more than 10 days, after the ^18^F-piflufolastat PET/CT. ^18^F-Flotufolastat (8 mCi [296 MBq] ± 20%) was administered as a single intravenous bolus per approved US prescribing information, and scans started approximately 60 minutes post-injection [8].

#### Quantitative image processing

Quantitative image processing for the bladder was performed using MIM Encore™ (MIM Software Inc., Cleveland, OH). The bladder activity of both ^18^F-piflufolastat and ^18^F-flotufolastat was quantitatively assessed by segmenting the bladder activity using VOIs. The initial segmentation was performed by a qualified image processing specialist before review and final editing by Reader 3 prior to sign-off. The initial VOI was drawn around the clearly present radioactive volume in the urinary bladder using a pre-specified, semi-automated spatial derivative tool (PETEdge, MIM Software Inc.) and edited if required to ensure it contained physiologic activity only, avoiding other organs and pathologic disease. The PET scan was used as the primary reference modality for drawing the bladder VOI, with the CT used as reference only in instances where no physiological activity was present or partial volume effects of adjacent uptake were seen.

### Study endpoints

The primary endpoint was the difference in bladder SUV_mean_ between ^18^F-piflufolastat and ^18^F-flotufolastat assessed in the bladder VOI. Secondary and tertiary endpoints included evaluation of additional quantitative bladder activity metrics (SUV_max_, SUV_peak_, and total radioactivity) for both ^18^F-piflufolastat and ^18^F-flotufolastat, the patient-level detection rate (percentage of patients with ≥1 PET-positive lesion out of all evaluable patients) for both ^18^F-piflufolastat and ^18^F-flotufolastat (overall and stratified by baseline PSA), and regional detection rates for ^18^F-piflufolastat and ^18^F-flotufolastat including for local recurrence in the prostate bed (vesicourethral anastomosis, retrovesical, and remnant seminal vesicles/lateral surgical margin subregions), in pelvic lymph nodes, and in extra-pelvic sites.

PET scans were interpreted by two independent readers who were dual board-certified in radiology and nuclear medicine and had prior clinical and research experience reading ^18^F-piflufolastat and ^18^F-flotufolastat scans. The readers were independently identified, contracted, and provided with study specific training by the imaging core laboratory. The readers were blinded to the study agent and all other clinical data. To reduce bias, there was a minimum 4-week period between reads of scans from the same patient, and the imaging core laboratory randomised the order in which the scans were presented to readers on a per-patient basis. The readers were trained to read scans for ^18^F-piflufolastat and ^18^F-flotufolastat per the respective independent SNMMI product-specific training (https://www.snmmilearningcenter.org) and US FDA-approved prescribing information [7, 8], and were instructed to identify lesions that they interpreted as positive for prostate cancer according to their training. Disagreements were resolved by a third independent reader (dual board-certified in radiology and nuclear medicine), allowing for adjudication by majority. Reader 3 was blinded to the results of the first two readers and performed a full, independent assessment of the scan. Majority read results are reported throughout.

### Safety

Safety was monitored throughout the study for both radiopharmaceuticals, and during a follow-up phone call 24 hours after the ^18^F-flotufolastat PET. Treatment-emergent adverse events (TEAEs) were defined per protocol as any adverse event (AE) that started or worsened after ^18^F-piflufolastat administration up to the follow-up safety monitoring call after ^18^F-flotufolastat administration. As ^18^F-piflufolastat was administered as part of routine clinical care, there was no post-^18^F-piflufolastat safety monitoring telephone call. AEs occurring after the study visit for ^18^F-piflufolastat administration were collected during Visit 3. AEs were not assessed for causality (i.e., related/not related) to ^18^F-piflufolastat by the site investigator, but were assigned as attributable to ^18^F-piflufolastat if the AE occurred from the administration of ^18^F-piflufolastat through the following day. Details of any AEs post-^18^F-flotufolastat were collected on the day of the ^18^F-flotufolastat PET/CT, and during the follow-up safety monitoring call that took place 24 hours afterwards. AEs were assessed for causality by the site investigator and were also assigned as attributable to ^18^F-flotufolastat if the AE occurred from the administration of ^18^F-flotufolastat through the following day.

All AEs were assessed and graded per The National Cancer Institute’s Common Terminology Criteria for AEs (CTCAE) version 5 [24], and were recorded in site source documents and individual patient electronic case report forms.

### Statistical analyses

Statistical analysis was completed after the electronic data capture database lock (31 July 2025), and all images were read and transferred following quality control checks by the independent imaging core laboratory (29 August 2025). The Sponsor was blinded to all imaging data until all imaging reads were complete, and data transferred.

Safety data were summarised descriptively for the safety analysis set, which comprised all patients who had received ^18^F-piflufolastat or ^18^F-flotufolastat.

The primary, secondary and tertiary endpoints were analysed using the efficacy analysis set which comprised all patients who received ^18^F-piflufolastat and ^18^F-flotufolastat, and who had evaluable PET scans for both radiopharmaceuticals. The primary endpoint was assessed for normality via histograms, quantile–quantile plots, and the Shapiro–Wilk test. The distribution of the difference in bladder SUV_mean_ between ^18^F-piflufolastat and ^18^F-flotufolastat was assessed as non-normal and the median of the differences between the paired values was evaluated using a Wilcoxon signed-rank test. To assess the impact of any missing primary endpoint data resulting from non-evaluable scans or dropouts after ^18^F-piflufolastat, two sensitivity analyses were conducted to examine the robustness of the primary analysis:

1. Assumed any missing ^18^F-flotufolastat bladder SUV_mean_ data were the same as the piflufolastat PET scan, aligning with the null hypothesis of no difference between ^18^F-piflufolastat and ^18^F-flotufolastat.
2. Assumed that any missing data were missing at random and analysed all available observations using a mixed-model repeated-measures (MMRM) approach.

Secondary endpoints were summarised descriptively, with 95% confidence intervals (CI) presented where appropriate.

## Results

### Patients

The study took place at nine sites in the US between October 2024 and June 2025.

**Fig. 2:**
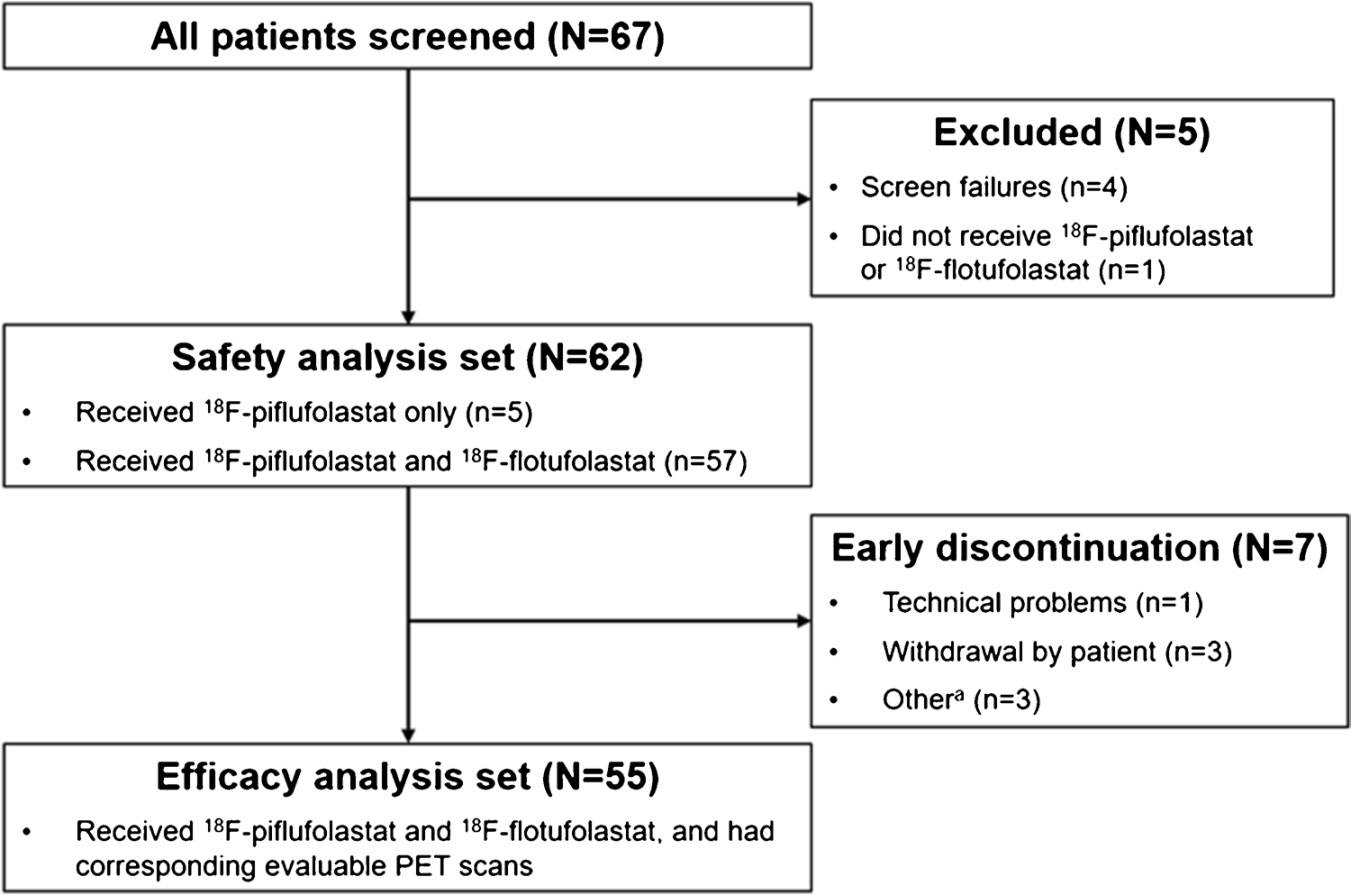
Flowchart of patient disposition. ^a^One patient missed their scheduled ^18^F-flotufolastat PET scan appointment; one patient did not have sufficient scan coverage of the bladder due to an urgent need to void mid-acquisition; and one patient was excluded by Investigators as they had undergone salvage therapy for the current episode of recurrence. ^18^F=Fluorine-18. PET = positron emission tomography

A total of 67 patients were screened (Fig. 2). Of these, 62 (92.5%) received either ^18^F-piflufolastat only (*n* = 5) or both ^18^F-piflufolastat and ^18^F-flotufolastat (*n* = 57) and were included in the safety analysis set. Seven patients from the safety analysis set were not included in the efficacy analysis set; five patients did not undergo ^18^F-flotufolastat PET, and two patients had scans that were not evaluable, as outlined in Fig. 2. Demographic and baseline characteristics for the safety analysis set and the efficacy analysis set are summarised in Table 1.

**Table 1:** Demographic and baseline characteristics

	Safety analysis set (*N =* 62)	Efficacy analysis set (*N* = 55)
Age, years		
Mean	67.8	67.9
SD	8.2	7.7
Range	39–85	39–83
Race, n (%)		
Asian	3 (4.8)	3 (5.5)
Black or African American	4 (6.5)	4 (7.3)
White	50 (80.6)	44 (80.0)
Other or not reported	5 (8.1)	4 (7.3)
ISUP Grade Group, n (%)		
1	3 (4.8)	3 (5.5)
2	21 (33.9)	18 (32.7)
3	24 (38.7)	21 (38.2)
4	3 (4.8)	3 (5.5)
5	11 (17.7)	10 (18.2)
Baseline BMI, kg/m^2^		
Median	28.2	28.2
Range	18–41	18–41
Time since prostate cancer diagnosis, months		
Median	54.8	52.3
Range	10–183	10–183
Prior therapies, n (%)		
RP only	56 (90.3)	50 (90.9)
RP with RT	2 (3.2)	1 (1.8)
RP plus other concomitant therapy	3 (4.8)	3 (5.5)
RP with RT plus other concomitant therapy	1 (1.6)	1 (1.8)
Baseline PSA, ng/mL		
Mean	0.27	0.28
SD	0.12	0.11
Range	0.01–0.50	0.09–0.50
PSA group, n (%)		
≤ 0.2 ng/mL	24 (38.7)	21 (38.2)
> 0.2 to 0.5 ng/mL	38 (61.3)	34 (61.8)

Data shown for evaluable patients

*BMI* body mass index; *ISUP* International Society of Urological Pathology; *PSA* prostate-specific antigen; *RP* radical prostatectomy, *RT* radiation therapy; *SD* standard deviation

### Bladder activity for ^18^F-piflufolastat and ^18^F-flotufolastat

**Fig. 3:**
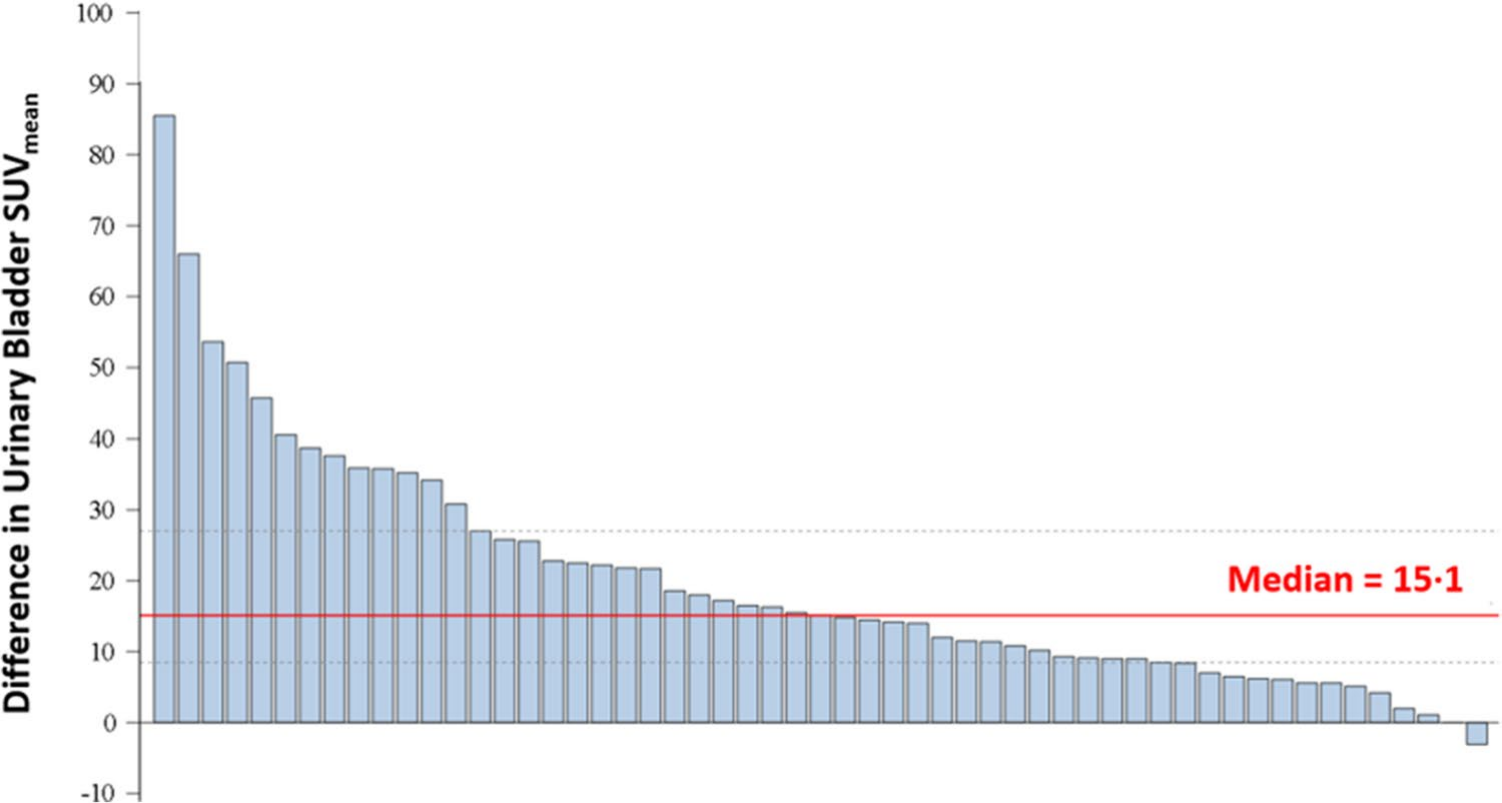
Waterfall plot of difference in urinary bladder SUV_mean_ (efficacy analysis set). Each bar represents data from one patient. A positive value represents a lower SUV_mean_ for ^18^F-flotufolastat

**Fig. 4:**
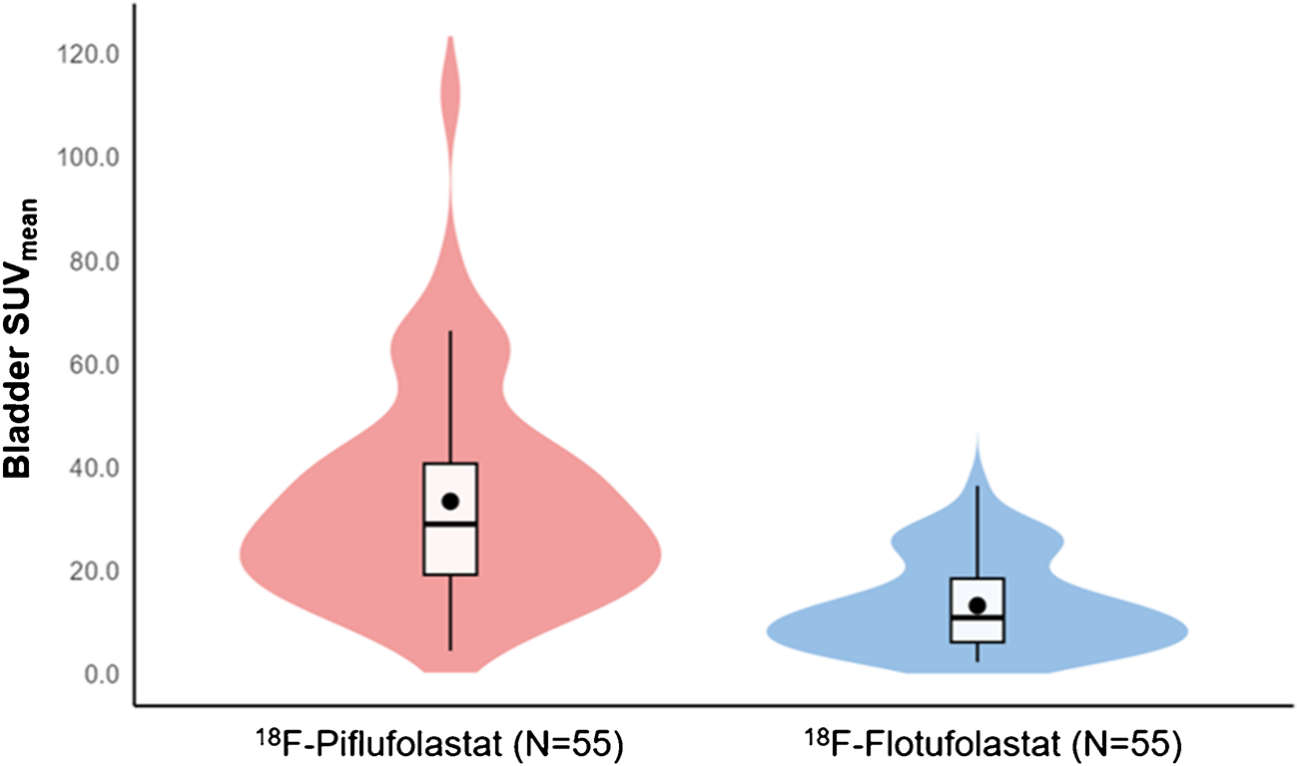
Violin plot showing quantitative assessment (SUV_mean_) of bladder activity for ^18^F-piflufolastat and ^18^F-flotufolastat (efficacy analysis set). Boxplots display the mean values (black dots), the median values (horizontal lines), IQR (box), and whiskers extend to 1.5 x IQR. The width of the violin indicates the density of the data points, with wider sections showing more data. ^18^F=Fluorine-18. IQR = interquartile range. SUV_mean_ = mean standardised uptake value

**Fig. 5:**
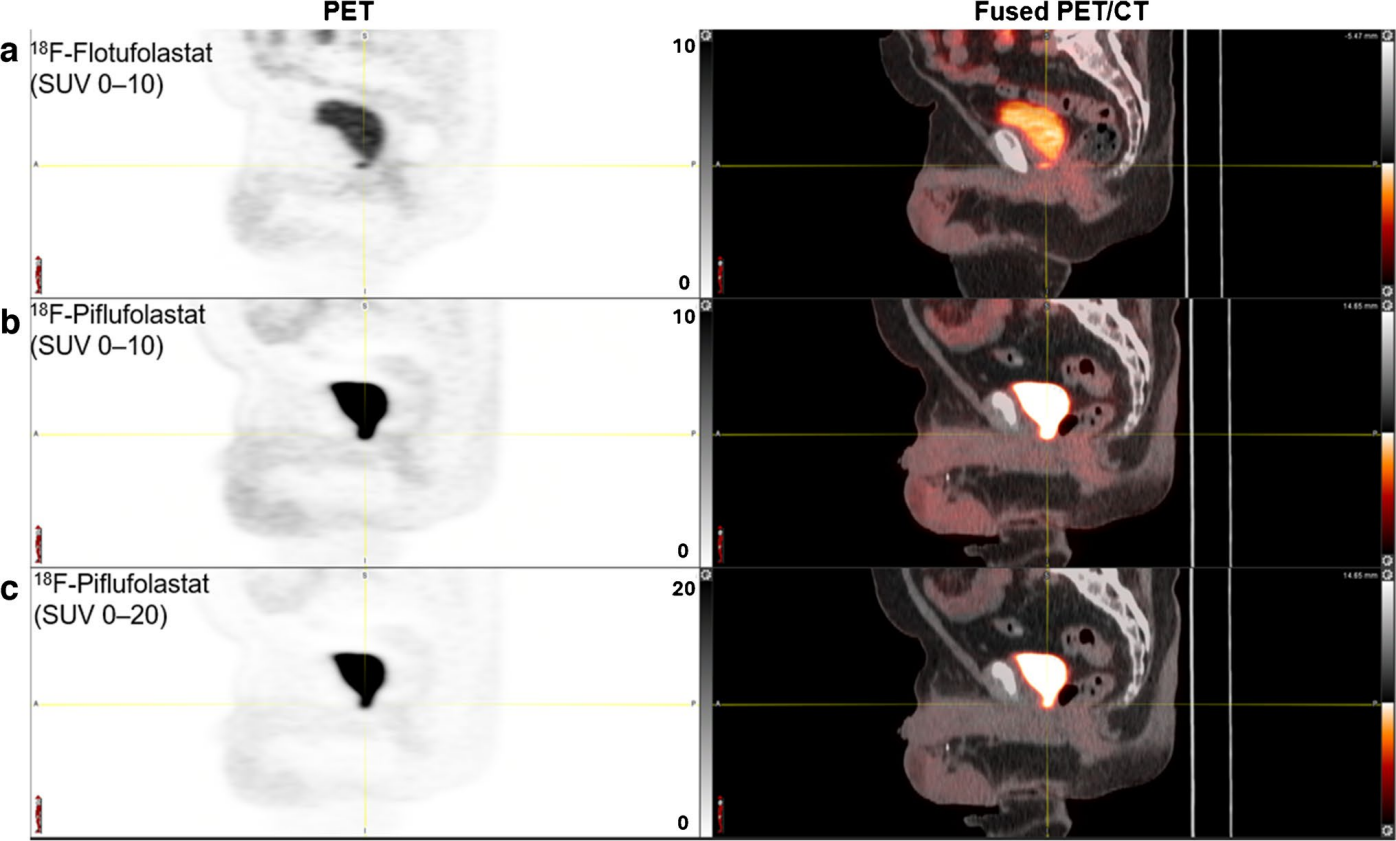
Example PET and fused PET/CT sagittal images from a patient with a baseline PSA level of 0.37 ng/mL who underwent ^18^F-piflufolastat PET and ^18^F-flotufolastat PET one day apart. ^18^F-Flotufolastat PET (row A; SUV scale, 0–10) identified a positive lesion in the vesicourethral anastomosis, which was not identified as separate from the bladder on ^18^F-piflufolastat PET on the same windowing (row B; SUV scale, 0–10), or with wider windowing at SUV 0–20 (row C). The bladder SUV_mean_ was 6.3 with ^18^F-flotufolastat PET and 40.5 with ^18^F-piflufolastat PET. ^18^F=Fluorine-18. CT = computed tomography. PET = positron emission tomography. PSA = prostate-specific antigen. SUV = standardised uptake value

^18^F-Flotufolastat had a lower bladder SUV_mean_ than ^18^F-piflufolastat in 96.4% (53/55) of patients (Fig. 3) and equal SUV_mean_ in one (1.8%) patient. ^18^F-Piflufolastat had a median bladder SUV_mean_ of 29.0 (interquartile range [IQR], 18.9–40.8). The median bladder SUV_mean_ for ^18^F-flotufolastat was significantly lower (10.9; IQR, 6.0–18.5), with a median difference of 15.1 (IQR, 8.5–27.0; *p* < 0.001) (Fig. 4). Example ^18^F-piflufolastat and ^18^F-flotufolastat PET/CT images from an enrolled patient are shown in Fig. 5.

Sensitivity analysis 1 (*n* = 62) was carried out where seven missing ^18^F-flotufolastat values were assumed equal to the patients’ ^18^F-piflufolastat values (i.e., it was assumed there was no difference in those patients). Using SUV_mean_ data for all 62 patients gave a median ^18^F-piflufolastat SUV_mean_ of 29.4 (IQR, 18.9–40.8); the median difference after the imputation of ^18^F-flotufolastat values was still statistically significant (14.1 [IQR, 6.1–25.6]; *p* < 0.001). Sensitivity analysis 2 (*n* = 62) was conducted using a MMRM where missing data were assumed missing at random, and maximum likelihood estimation was used to fit model parameters. The analysis showed a statistically significant difference in the means (20.1; 95% CI, 15.5–24.7; *p* < 0.001).

The evaluation of further quantitative bladder metrics showed the total bladder radioactivity, SUV_max_, and SUV_peak_ to all be significantly lower with ^18^F-flotufolastat than with ^18^F-piflufolastat (all *p* < 0.001; Supplementary Table 1), although no adjustment for multiplicity was carried out for these exploratory analyses.

### Patient-level detection rates

**Fig. 6:**
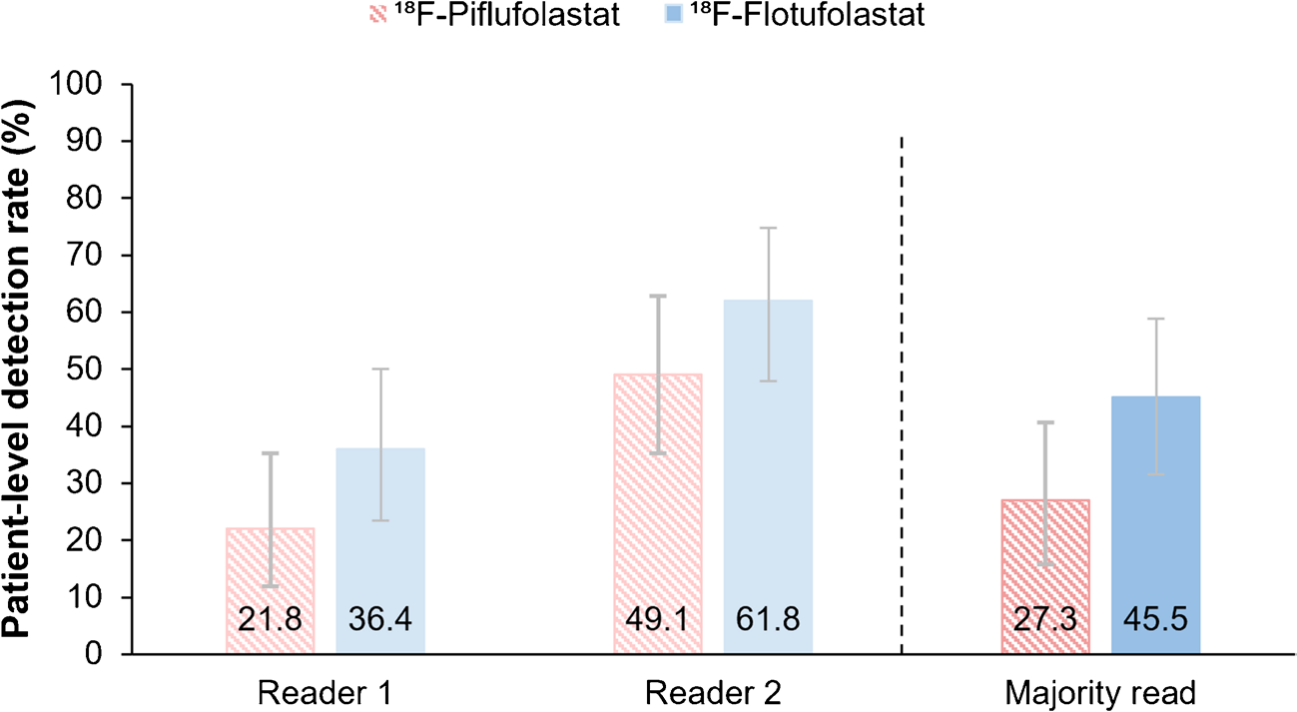
Patient-level detection rates for ^18^F-piflufolastat and ^18^F-flotufolastat in the efficacy analysis population, by reader and by majority read. *N* = 55. Error bars show 95% confidence intervals. ^18^F=Fluorine-18

Overall, 27.3% (15/55; 95% CI, 16.1–41.0) of patients had ^18^F-piflufolastat-positive lesions, and 45.5% (25/55; 95% CI, 32.0–59.4) had ^18^F-flotufolastat-positive lesions by majority read (Fig. 6). All ^18^F-piflufolastat scans considered positive by the blinded majority read were also considered positive on ^18^F-flotufolastat PET. In addition, by majority read, ^18^F-flotufolastat identified lesions for an additional 10 (18.2%) patients who had a negative ^18^F-piflufolastat scan.

**Fig. 7:**
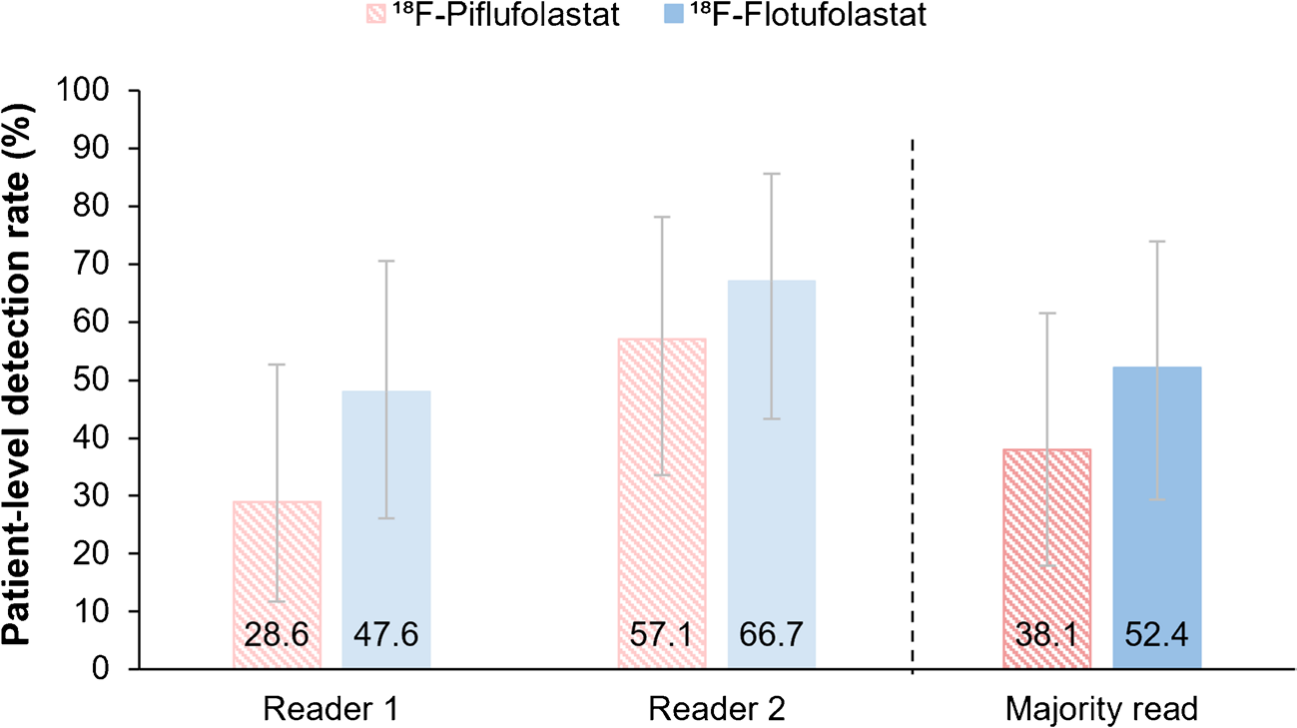
Patient-level detection rates for ^18^F-piflufolastat and ^18^F-flotufolastat in patients with very low PSA levels (≤0.2 ng/mL), by reader and by majority read. *N* = 21. Error bars show 95% confidence intervals. ^18^F=Fluorine-18

A secondary endpoint analysis among the small subset of patients with very low PSA levels (≤0.2 ng/mL; *N* = 21) showed the patient-level detection rate by majority read to be 38.1% (8/21; 95% CI, 18.1–61.6) for ^18^F-piflufolastat and 52.4% (11/21; 95% CI, 29.8–74.3) for ^18^F-flotufolastat (Fig. 7).

### Region-level detection rates

#### Prostate bed

**Fig. 8:**
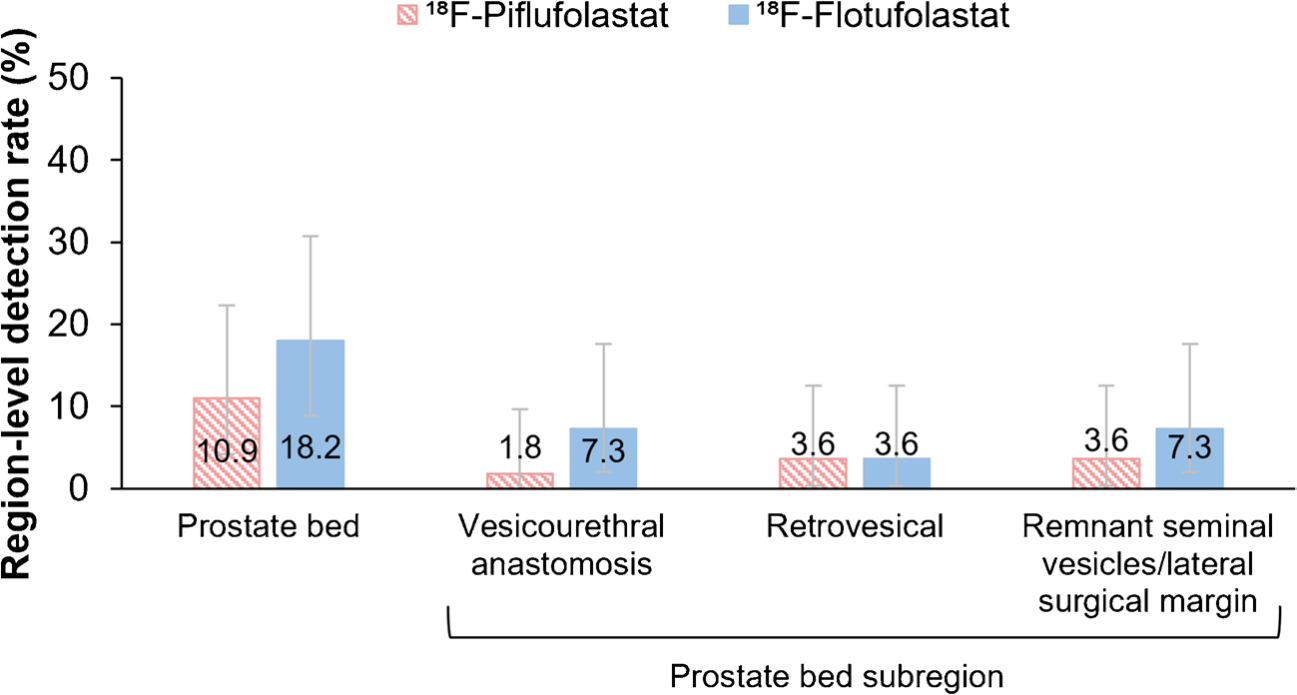
Prostate bed region-level ^18^F-piflufolastat and ^18^F-flotufolastat detection rates by majority read. *N* = 55. Error bars show 95% confidence intervals. ^18^F=Fluorine-18

In total, 10.9% (6/55; 95% CI, 4.1–22.2) of patients had a positive ^18^F-piflufolastat scan in the prostate bed region, and 18.2% (10/55; 95% CI, 9.1–30.9) had a positive ^18^F-flotufolastat scan in the prostate bed region. Figure 8 presents the detection rates in the prostate bed stratified by subregion. In addition to the 10.9% (6/55) of patients who had a positive finding in the prostate bed region on both ^18^F-piflufolastat and ^18^F-flotufolastat PET, a further four patients (7.3%) had a positive finding in the prostate bed region identified on ^18^F-flotufolastat PET only (Supplementary Table 2).

#### Pelvic lymph nodes

In total, 14.5% (8/55; 95% CI, 6.5–26.7) of patients had ^18^F-piflufolastat-positive lesions, and 16.4% (9/55; 95% CI, 7.8–28.8) of patients had ^18^F-flotufolastat-positive lesions in pelvic lymph nodes. Overall, 7/55 patients (12.7%) had a positive finding in pelvic lymph nodes on ^18^F-piflufolastat and ^18^F-flotufolastat PET (majority read). Two additional patients (3.6%) had positive pelvic lymph node findings on ^18^F-flotufolastat PET but not on ^18^F-piflufolastat PET, and a single patient (1.8%) had a positive pelvic lymph node on ^18^F-piflufolastat PET but not on ^18^F-flotufolastat PET.

#### Other (extra-pelvic) sites

Detection rates in extra-pelvic sites were 7.3% (4/55; 95% CI, 2.0–17.6) for ^18^F-piflufolastat and 21.8% (12/55; 95% CI, 11.8–35.0) for ^18^F-flotufolastat.

### Safety

There were no significant safety concerns for either ^18^F-piflufolastat or ^18^F-flotufolastat (Appendix; Supplementary Table 3).

## Discussion

The advent of highly sensitive PSMA-PET has facilitated the accurate localisation of recurrent prostate cancer lesions at low (≤0.5 ng/mL) PSA levels [18, 25, 26]. However, the potential for high levels of urinary radioactivity from some of these renally cleared radiopharmaceuticals can obscure lesions in the prostate/prostate bed and surrounding areas [11–13], which may confound image interpretation. Here, we present data from the first intra-patient comparison of urinary radioactivity of ^18^F-piflufolastat and ^18^F-flotufolastat PET in men with post-prostatectomy BCR of prostate cancer.

Previous SUV estimates have indicated lower urinary radioactivity for ^18^F-flotufolastat (median SUV_mean_, 12.5; median SUV_max_, 17.1) [19] than published in separate studies for ^18^F-piflufolastat (median SUV_max_, 61.7 to 79.3) [10, 14]. This was confirmed in the present intra-patient comparison study. In our study, patients underwent ^18^F-piflufolastat and ^18^F-flotufolastat PET a maximum of 10 days apart and we show there to be a significantly lower urinary bladder SUV_mean_ with ^18^F-flotufolastat than ^18^F-piflufolastat (median SUV_mean_, 10.9 versus 29.0, respectively; *p* < 0.001) as well as SUV_max_ (median bladder SUV_max_, 18.1 versus 54.7, respectively; *p* < 0.001). The significant difference we observed in bladder activity has the potential to impact clinical practice in several ways. As noted above, bladder activity may potentially impact lesion detectability, especially where peri-vesical lesion avidity is similar to or lower than activity in the adjacent bladder, which, as illustrated in Fig. 5, image windowing cannot fully remedy. This is notwithstanding that the typical approach to clinical interpretation for oncology PET (both PSMA and non-PSMA) involves the routine use of an upper SUV threshold not exceeding 10. Furthermore, while not measured in this study, the potential of bladder activity to impact lesion detectability is anticipated to inherently influence reader confidence, particularly in ruling out or positively calling prostate bed or peri-ureteric disease.

Among this population of patients with PSA levels ≤0.5 ng/mL, 38.2% of patients had a PSA level ≤ 0.2 ng/mL, which is broadly considered to be below the current threshold for defining post-prostatectomy recurrence based on conventional imaging [2]. Despite this, clinically meaningful detection rates were observed with both radiopharmaceuticals, with ^18^F-flotufolastat identifying lesions for an additional 18.2% of patients compared with ^18^F-piflufolastat PET. It should be noted that PET-positive findings were not verified in this study for either radiopharmaceutical. Notwithstanding the FDA-approved status of both radiopharmaceuticals at time of study, as the previously reported histopathologically verified patient-level positive predictive values for ^18^F-piflufolastat and ^18^F-flotufolastat are similar [27], the higher rate of detection with ^18^F-flotufolastat than with ^18^F-piflufolastat observed in our study can be expected to correlate with a higher rate of true positive lesions.

Consistent with existing data in the literature, we observed higher overall detection rates with ^18^F-flotufolastat than with ^18^F-piflufolastat [27]. Previous data from the SPOTLIGHT study in patients with BCR after curative-intent therapy show ^18^F-flotufolastat to have a detection rate of 64% (77/121) among patients with a PSA level < 0.5 ng/mL [18], higher than the rate observed in our study (45.5%) which included a larger proportion of patients with very low PSA levels. The SPOTLIGHT study recruited just three patients with a PSA level < 0.2 ng/mL, for whom a detection rate of 33% was observed [25]. Here, a secondary endpoint analysis among the 21 enrolled patients with a PSA level ≤ 0.2 ng/mL shows a higher ^18^F-flotufolastat detection rate of 52.4%. Prior data reported from the Phase 3 CONDOR trial, which included no patients with a PSA level < 0.2 ng/mL, show the ^18^F-piflufolastat detection rate at PSA levels <0.5 ng/mL to be 36% (majority read among 69 patients) [22], which is higher than the 27.3% observed for ^18^F-piflufolastat among the present very low PSA cohort.

Taken together with our secondary findings, these data suggest that, while both ^18^F-piflufolastat and ^18^F-flotufolastat can provide clinically meaningful localisation of lesions early in the recurrence timeline, ^18^F-flotufolastat may yield improved detection rates overall. Furthermore, these data highlight the potential need for revisions to the definitions of BCR (which are currently based on conventional imaging modalities) to account for the greater sensitivity of PSMA-targeted PET radiopharmaceuticals.

It is well documented that initiation of salvage radiation therapy (SRT) at lower PSA levels improves patient outcomes [28]. PET-guided SRT has been demonstrated to significantly improve biochemical-free survival compared with SRT guided by conventional imaging [29] and preliminary data that suggest use of PSMA-targeted PET radiopharmaceuticals for SRT planning has potential to improve patient outcomes [30, 31] warrant further exploration. Our data show ^18^F-flotufolastat provides high detection rates at very low PSA levels, and may offer improved detection in the prostate bed, likely due to its significantly lower urinary activity. Together, these findings suggest that ^18^F-flotufolastat may provide optimal guidance of SRT in patients with early BCR of prostate cancer.

There are some limitations to the present analysis. First, while our study provides a quantitative comparison of SUVs in the bladder between ^18^F-piflufolastat and ^18^F-flotufolastat, the data were not powered to assess if lesions had been obscured by bladder activity with either radiopharmaceutical. Thus, we did not prospectively evaluate the SUVs of metastatic lesions in comparison with bladder SUVs, and this could be the focus of future work. Second, although the lack of randomisation in the order of scans might be considered a limitation, all scans were reviewed centrally by independent readers in a fully randomised order to minimise the potential for sequence bias on the study outcomes. Third, as discussed above, additional histopathological or imaging verification of PET-positive lesions was beyond the scope of the present study. Fourth, while the data may suggest superior diagnostic performance of ^18^F-flotufolastat than ^18^F-piflufolastat, the study was not powered to evaluate differences in detection rates. Fifth, some differences in the detection rates between the two readers were observed, however, both readers consistently reported a higher detection rate for ^18^F-flotufolastat than for ^18^F-piflufolastat. The use of a third reader to provide adjudication reads was a particular strength of our study. This third reader allowed for detection rates presented by majority, that while being between that of Reader 1 and 2, trended closer to Reader 1. Finally, while ^18^F-flotufolastat may demonstrate low bladder activity and high detection rates that might inform future treatment, the impact on clinical decision making, salvage therapy planning, and long-term outcomes were not measured. Thus, while the impact on patients is unknown, it can be inferred that positive findings in patients with BCR are inherently more actionable, with previous data reporting changes to management plans for 63–79% of patients following ^18^F-flotufolastat PET [32, 33].

## Conclusions

This intra-patient comparator study confirms existing data by showing that ^18^F-flotufolastat has significantly lower urinary radioactivity than ^18^F-piflufolastat, which may help to optimise image assessment in regions close to the urinary tract, as supported in the present study by the higher proportion of patients observed to have ^18^F-flotufolastat-positive lesions overall, especially in the prostate bed region, compared with ^18^F-piflufolastat-positive lesions. Furthermore, ^18^F-flotufolastat-positive lesions were detected in more than half of all patients with a PSA level below the currently accepted PSA threshold (≤0.2 ng/mL) for defining BCR.

## Supplementary information

ESM 1 (PDF 2160 kb)

## Data Availability

The data that support the findings of this study are available from the corresponding author upon reasonable request.
