# Supplementary material for: An intra-patient contemporaneous comparison of 18F-piflufolastat and 18F-flotufolastat urinary radioactivity and pelvic region detection rates in men with low PSA biochemical recurrence of prostate cancer after radical prostatectomy
Source: Eur J Nucl Med Mol Imaging. 2026 Feb 23;53(6):3529–41. doi: 10.1007/s00259-025-07732-y (PMC13121232; doi:10.1007/s00259-025-07732-y)
Supplement: Supplementary file 1 — (PDF 2160 kb) [file 259_2025_7732_MOESM1_ESM.pdf]

Supplementary appendix: An intra-patient contemporaneous comparison of ^{18}F-piflufolastat and ^{18}F-flotufolastat urinary radioactivity and pelvic region detection rates in men with low PSA biochemical recurrence of prostate cancer after radical prostatectomy

Contents

1. Additional quantitative measures of bladder radioactivity for ^{18}F-piflufolastat and ^{18}F-flotufolastat......2

2. Comparison of positive and negative prostate bed scan results with ^{18}F-piflufolastat and ^{18}F-flotufolastat......2

3. Summary of adverse events (AE)......3

4. Study protocol......4

1. Additional quantitative measures of bladder radioactivity for ^{18}F-piflufolastat and ^{18}F-flotufolastat

Supplementary table 1 Total bladder radioactivity, SUV_{max}, and SUV_{peak} bladder radioactivity for ^{18}F-piflufolastat and ^{18}F-flotufolastat

	**^{18}F-Piflufolastat (N=55)**	**^{18}F-Flotufolastat (N=55)**	**Difference (^{18}F-Piflufolastat versus ^{18}F-Flotufolastat)**	**p-value**
**Median (IQR) total bladder radioactivity, MBq**	164.5 (93.0–199.1)	45.8 (24.1–62.0)	88.9 (57.0–140.1)	<0.001
**Median (IQR) SUV_{max}**	54.7 (30.5–117.0)	18.1 (10.5–38.0)	32.0 (13.2–67.5)	<0.001
**Median (IQR) SUV_{peak}**	52.9 (28.6–97.3)	16.7 (8.8–29.9)	30.2 (13.6–59.5)	<0.001

^{18}F=Fluorine-18. IQR=interquartile range. SUV=standardised uptake value. SUV_{max}=maximum standardised uptake value. SUV_{peak}=peak standardised uptake value.

2. Comparison of positive and negative prostate bed scan results with ^{18}F-piflufolastat and ^{18}F-flotufolastat

Supplementary table 2 Comparison of positive and negative scan results with ^{18}F-piflufolastat and ^{18}F-flotufolastat in the prostate bed region and subregions by majority read

**Region/subregion**		**^{18}F-Piflufolastat** PET	
	**^{18}F-Flotufolastat PET**	Positive, n (%)	Negative, n (%)
**Prostate bed**	Positive, n (%)	6 (10.9)	4 (7.3)
	Negative, n (%)	0	45 (81.2)
**Vesicourethral anastomosis**	Positive, n (%)	1 (1.8)	3 (5.5)
	Negative, n (%)	0	51 (92.7)
**Retrovesical**	Positive, n (%)	1 (1.8)	1 (1.8)
	Negative, n (%)	1 (1.8)	52 (94.5)
**Remnant seminal vesicles/ Lateral surgical margin**	Positive, n (%)	2 (3.6)	2 (3.6)
	Negative, n (%)	0	51 (92.7)

N=55.
^{18}F=Fluorine-18.

3. Summary of adverse events (AE)

Overall, 8.1% (5/62) of patients in the safety analysis set experienced a total of seven treatment emergent AE (TEAE). In total, 4.8% (3/62) of patients who received ^{18}F-piflufolastat experienced four AE attributable to ^{18}F-piflufolastat and 3.5% (2/57) of patients who received ^{18}F-flotufolastat experienced three AE attributable to ^{18}F-flotufolastat. Three AE attributable to ^{18}F-piflufolastat (flushing, fatigue, and diarrhoea [all n=1]) were classed as mild (Common Terminology Criteria for Adverse Events [CTCAE] Grade 1), and one patient had one serious AE (CTCAE Grade 3 dyspnoea exertional) that was attributable to ^{18}F-piflufolastat. All AE attributable to ^{18}F-flotufolastat (abdominal discomfort, diarrhoea, and taste disorder [all n=1]) were classed as mild (CTCAE Grade 1). There were no serious AE attributable to ^{18}F-flotufolastat.

Supplementary table 3 Summary of AE data

**AE by system organ class preferred term, n (%)**	**AE attributable to ^{18}F-piflufolastat (N=62)**	**AE attributable to ^{18}F-flotufolastat (N=57)**
**Gastrointestinal disorders**		
Abdominal discomfort	0	1 (1.8)
Diarrhoea	1 (1.6)	1 (1.8)
**General disorders and administration site conditions**		
Fatigue	1 (1.6)	0
**Nervous system disorders**		
Taste disorder	0	1 (1.8)
**Respiratory, thoracic, and mediastinal disorders**		
Dyspnoea exertional	1 (1.6)*	0
**Vascular disorders**		
Flushing	1 (1.6)	0

All AEs are CTCAE Grade 1, unless otherwise specified.
*CTCAE Grade 3 AE.
^{18}F=Fluorine-18. AE=adverse event. CTCAE=Common Terminology Criteria for Adverse Events.

4. Study protocol

Note, the amendment to introduce blinding of readers to the study agent took place post-finalisation of the study protocol.

CLINICAL STUDY PROTOCOL

Intra-patient Comparison of Urinary Radioactivity Following Piflufolastat (^{18}F) and Flotufolastat (^{18}F) PET in Men with Low PSA Biochemical Recurrence of Prostate Cancer Following Radical Prostatectomy

BED-PSMA-411

Phase: 4

IND Number: 141561

Protocol Version and Date: Protocol Version 4, 22-Apr-2025

Sponsor: Blue Earth Diagnostics

Confidentiality Statement

This confidential information about an investigational product is provided for the exclusive use of investigators of this product and is subject to recall at any time. The information in this document may not be disclosed unless applicable laws or regulations require such disclosure. Subject to the foregoing, this information may be disclosed only to those persons involved in the study who have a need to know, with the obligation not to further disseminate this information.

Conflicts of Interest

Blue Earth Diagnostics Ltd is responsible for the manufacture of flotufolastat (^{18}F) injection.

Blue Earth Diagnostics

Clinical Study Protocol

Intra-patient Comparison of Urinary Radioactivity Following Piflufolastat (^{18}F) and Flotufolastat (^{18}F) PET in Men with Low PSA Biochemical Recurrence of Prostate Cancer Following Radical Prostatectomy

Protocol Number:	BED-PSMA-411
Protocol Version / Date:	Protocol Version 4, 22-Apr-2025
Investigational Product:	Flotufolastat (^{18}F) injection (Trade name: POSLUMA®)
IND Number:	141561
Development Phase:	Phase 4
Sponsor:	Blue Earth Diagnostics [redacted]
Funding Organization:	Blue Earth Diagnostics
Coordinating Investigator:	Name: [redacted]
Medical Monitor:	Name: [redacted]
Coordinating Center:	[redacted]

Approval:

Sponsor Signature ([redacted]
VP, Clinical Science & Medical Affairs Advisor)

Date

TABLE OF CONTENTS

TABLE OF CONTENTS 3

LIST OF TABLES 6

LIST OF FIGURES 6

LIST OF ABBREVIATIONS 7

PROTOCOL SYNOPSIS 9

STUDY SCHEMA 12

1 BACKGROUND 13

1.1 Overview of Non-clinical Studies 13

1.2 Overview of Clinical Studies 14

1.3 Urinary Radioactivity 15

2 STUDY RATIONALE 18

2.1 Risk-Benefit Assessment 18

2.1.1 Benefits 19

2.1.2 Risks 19

3 STUDY OBJECTIVES 20

3.1 Primary Objective 20

3.2 Secondary Objectives 20

3.3 Tertiary Objectives 20

3.4 Safety Objectives 20

4 STUDY DESIGN 21

4.1 Study Overview 21

5 CRITERIA FOR EVALUATION 22

5.1 Primary Endpoint 22

5.2 Secondary Endpoints 22

5.3 Tertiary Endpoints 22

5.4 Safety Evaluations 22

6 PATIENT SELECTION 23

6.1 Study Population 23

6.2 Inclusion Criteria 23

6.3 Exclusion Criteria 23

7 CONCOMITANT MEDICATIONS 24

7.1 Allowed Medications and Treatments 24

7.2 Prohibited Medications and Treatments 24

8 STUDY TREATMENTS 25

8.1 Method of Assigning Patients to Treatment Groups 25
8.2 Blinding 25
8.3 Formulation of the Investigational Product 25
8.4 Formulation of Comparator 26
8.5 Packaging and Labeling 26
8.5.1 Flotufolastat (^{18}F) Injection 26
8.5.2 Piflufolastat (^{18}F) Injection 26
8.6 Radiopharmaceutical Supply at the Site 26
8.7 Dosage/Dosage Regimen 27
8.8 Dispensing 27
8.9 Administration Instructions 27
8.10 Storage 27
8.11 Investigational Product Accountability 28
8.12 Measures of Treatment Compliance 28
8.13 Primary Study Completion and Participant Completion Date 28
9 STUDY PROCEDURES AND GUIDELINES 29
9.1 Clinical Assessments 31
9.1.1 Concomitant Medications 31
9.1.2 Demographic and Baseline Characteristics 31
9.1.3 Medical History 31
9.1.4 Imaging Studies 31
9.1.5 Physical Examination 31
9.1.6 Vital Signs 31
9.1.7 PSA Levels 31
9.2 Clinical Laboratory Measurements 31
10 EVALUATIONS BY VISIT 32
10.1 Visit 1 – Screening (Days -14 to 1) 32
10.2 Visit 2 – Scan 1 (Day 1) 32
10.3 Visit 3 – Scan 2 (Days 2 to 10) 32
10.4 Visit 4 – 24 Hours After Scan 2 (Days 3 to 11; Telephone) 33
11 IMAGING PROTOCOL 34
11.1 PET Scanner 34
11.2 Injection Administration 34
11.3 PET/CT Acquisition 34
11.4 Image Transfer 35
11.5 Image Analysis 35
12 ADVERSE EVENT REPORTING AND DOCUMENTATION 36
12.1 Adverse Events 36
12.2 Adverse Event Severity 37

12.3 Adverse Event Relationship to Investigational Product 38
12.4 Serious Adverse Events 38
12.4.1 Serious Adverse Event Reporting 38
12.4.2 Suspected Unexpected Serious Adverse Reactions 39
12.5 Medical Monitoring 39
13 DISCONTINUATION AND REPLACEMENT OF PATIENTS 40
13.1 Discontinuation/Withdrawal From the Study 40
13.2 Additional Patients 40
13.3 Lost to Follow-up 40
14 PROTOCOL VIOLATIONS 41
15 STATISTICAL METHODS AND CONSIDERATIONS 42
15.1 Estimation of Sample Size 42
15.2 Data Sets to be Analyzed 42
15.3 Demographic and Baseline Characteristics 42
15.4 Analysis of Primary Endpoint(s) 42
15.5 Analysis of Secondary and Tertiary Endpoint(s) 43
15.6 Planned Interim Analysis 43
16 DATA COLLECTION, RETENTION AND MONITORING 44
16.1 Data Collection Instruments 44
16.2 Data Management Procedures 44
16.3 Data Quality Control and Reporting 44
16.4 Archiving of Data 45
16.5 Availability and Retention of Investigational Records 45
16.6 Monitoring and Auditing 45
16.7 Patient Confidentiality 45
17 REGULATORY, ETHICAL AND STUDY OVERSIGHT CONSIDERATIONS 47
17.1 Institutional Review Boards and Independent Ethics Committees 47
17.2 Amendments 47
17.3 Patient Information and Consent 47
17.4 Post-trial Care 48
17.5 Publications 48
17.6 Investigator Responsibilities 48
18 REFERENCES 50
APPENDIX 1. PROTOCOL AGREEMENT 52

LIST OF TABLES

Table 1: Schedule of Study Procedures 30
Table 2: Treatment-emergent Adverse Event Definitions 36
Table 3: CTCAE (V5) AE Severity Grading 37
Table 4: AE Severity Grading 37
Table 5: AE Relationship to IP (Flotufolastat (^{18}F)) 38

LIST OF FIGURES

Figure 1: Overall Study Design 12
Figure 2: Quantitative Assessment of Bladder Radioactivity: SUV_{max} and SUV_{mean} 16
Figure 3: Quantitative Analysis of SUV_{max} and SUV_{mean} for Flotufolastat (^{18}F) Compared to Other Renally Cleared PSMA PET Ligands 17
Figure 4: Structural Formula of Flotufolastat (^{18}F) Injection 25
Figure 5: Structural Formula of Piflufolastat (^{18}F) Injection 26

LIST OF ABBREVIATIONS

Abbreviation	Definition
^{18}F	Fluorine-18
AE	Adverse event
AUC	Area under the concentration time curve
AUC_{0-t}	Area under the concentration time curve from hour 0 to the time of last measurable concentration
AUC_{0-24}	Area under the concentration time curve from hour 0 to 24 hours post-dose
BCR	Biochemical recurrence
BED	Blue Earth Diagnostics
CT	Computed tomography
CTCAE	Common Terminology Criteria for Adverse Events
CYP	Cytochrome P450
EAS	Efficacy Analysis Set
eCRF	Electronic case report form
EDC	Electronic Data Capture
EU	European Union
FAS	Full Analysis Set
FDA	Food and Drug Administration
GCP	Good Clinical Practice
HIPAA	Health Insurance Portability and Accountability Act of 1996
IB	Investigator's Brochure
ICF	Informed Consent Form
ICH	International Council for Harmonization
IEC	Independent Ethics Committee
IP	Investigational Product
IRB	Institutional Review Board
IV	Intravenous
MBq	MegaBecquerel
mCi	MilliCurie
MedDRA	Medical Dictionary for Regulatory Activities
µ/mSv	Micro/milliSievert
PET	Positron Emission Tomography
PLN	Pelvic lymph node
PPS	Per Protocol Set
PSA	Prostate-specific antigen
PSMA	Prostate-specific membrane antigen

Abbreviation	Definition
rh	Radiohybrid
RP	Radical prostatectomy
SAE	Serious adverse event
SAF	Full Safety Set
SAP	Statistical Analysis Plan
SiFA	Silicon-fluorine acceptor
SUSAR	Suspected unexpected serious adverse reaction
SUV	Standardized uptake value
SUV_{max}	Maximum SUV
SUV_{mean}	Mean SUV
SUV_{peak}	Peak SUV
TEAE	Treatment-emergent adverse event
US	United States
USP	United States Pharmacopeia
USPI	United States Prescribing Information

PROTOCOL SYNOPSIS

Study Title	Intra-patient Comparison of Urinary Radioactivity Following Piflufolastat (^{18}F) and Flotufolastat (^{18}F) PET in Men with Low PSA Biochemical Recurrence of Prostate Cancer Following Radical Prostatectomy
Phase	Phase 4
Sponsor	Blue Earth Diagnostics (BED)
Funding Organization	BED
Study Design	This is a multi-center, prospective intra-patient comparator study of urinary radioactivity (standardized uptake values [SUV]) following both piflufolastat (^{18}F) and flotufolastat (^{18}F) positron emission tomography (PET) in patients with low prostate-specific antigen (PSA; ≤0.5 ng/mL) biochemical recurrence (BCR) of prostate cancer following radical prostatectomy (RP).<br>Each patient will be administered a single dose of piflufolastat (^{18}F) on Day 1, followed by a PET scan per standard institutional practice and in line with the PYLARIFY United States Prescribing Information (USPI). At least 24 hours after the piflufolastat (^{18}F) scan, but within 10 calendar days, all patients will be administered a single dose of flotufolastat (^{18}F) followed by a PET scan. Each patient will receive both radiopharmaceuticals and, thus, serve as their own control.<br>For the primary endpoint analysis, volumes of interest (VOIs) will be placed over the urinary bladder, and radioactivity (SUV metrics) will be assessed following administration of each radiopharmaceutical.<br>For secondary and tertiary endpoints, both the piflufolastat (^{18}F) and flotufolastat (^{18}F) PET scans will be read by two independent nuclear medicine physicians or nuclear radiologists. They will be blinded to the patient's clinical history but will have been trained to read scans for each respective radiopharmaceutical, using product-specific training and in line with the Food and Drug Administration (FDA) approved USPI for the respective radiopharmaceutical. To facilitate use of the appropriate product-specific training, the independent readers will be unblinded to the administered agent. Any image interpretation disagreements will be resolved by a third independent adjudicator.
Study Rationale	Data from the BED-sponsored Phase 1 and Phase 3 flotufolastat (^{18}F) injection FDA marketing authorization studies demonstrate relatively low urinary radioactivity, on average, for flotufolastat (^{18}F) compared to those published for other approved renally cleared radiopharmaceuticals used for prostate-specific membrane antigen (PSMA) PET, including piflufolastat (^{18}F). This may represent a clinically meaningful difference for flotufolastat (^{18}F) given the challenge of delineating local recurrences (lesions within and adjacent to the prostate bed) of prostate cancer at low PSA values. Direct comparison of urinary radioactivity for flotufolastat (^{18}F) to another FDA approved- ^{18}F-labeled radiopharmaceutical for PSMA PET, (piflufolastat (^{18}F)), in a head to--head study in patients with low PSA (≤0.5 ng/mL) BCR of prostate cancer following RP, will likely provide valuable information on this biodistribution topic.<br>In addition, the secondary and tertiary objectives of the study will provide supportive data for the two radiopharmaceuticals, with respect to detection rates and other organ biodistribution metrics in this population of patients with low PSA BCR of prostate cancer.

Primary Objectives and Endpoints	**Objective:**<br>• To compare urinary bladder radioactivity observed on piflufolastat (^{18}F) PET and flotufolastat (^{18}F) PET.	**Endpoint:**<br>• Difference in urinary bladder mean SUV (SUV_{mean}) between piflufolastat (^{18}F) and flotufolastat (^{18}F).
Secondary Objectives and Endpoints	**Objective:**<br>To assess the following for piflufolastat (^{18}F) PET and flotufolastat (^{18}F) PET:<br>• Detection rates;<br>• Detection rates stratified by PSA level;<br>• Prostate bed detection rates: local recurrences by subregion;<br>• Pelvic lymph node (PLN) detection rates.	**Endpoint:**<br>Estimate of the following for each radiopharmaceutical:<br>• Patient-level detection rate;<br>• Patient-level detection rate by baseline PSA categories;<br>• Detection rate for local recurrences in prostate bed for the following subregions:<br>o vesicourethral anastomosis lesions;<br>o retrovesical lesions;<br>o remnant seminal vesicles/lateral surgical margin lesions;<br>• Detection rate for PLN lesions.
Tertiary Objectives and Endpoints	**Objective:**<br>To assess the following for piflufolastat (^{18}F) PET and flotufolastat (^{18}F) PET:<br>• Ureteric radioactivity;<br>• Bladder radioactivity;<br>• Physiological radioactivity;<br>• Extra-pelvic detection rates;	**Endpoint:**<br>Estimate of the following for each radiopharmaceutical:<br>• Central reader qualitative assessment of the presence or absence of ureteric radioactivity;<br>• Maximum SUV (SUV_{max}), peak SUV (SUV_{peak}) and total bladder radioactivity;<br>• SUV_{mean}, SUV_{peak} for normal organs, including liver and spleen;<br>• Detection rate for distant metastatic lesions and in the following subregions:<br>o bone lesions;<br>o extra-pelvic lymph node lesions;<br>o extra-pelvic soft tissue lesions;

	• Qualitative analysis of urinary radioactivity interference with image assessment.	• Qualitative 3-point scale by expert readers (as previously described by Kuo et al, 2024).
Safety	Safety will be evaluated by the incidence of treatment-emergent adverse events (TEAEs) after each PSMA PET scan.	
Study Sites	Five to 10 centers in the United States (US)	
Investigational Product	Flotufolastat (^{18}F) injection (Trade name: POSLUMA®)	
Comparator Product	Piflufolastat (^{18}F) injection (Trade name: PYLARIFY®)	
Study and Patient Duration	Study Duration: 6 to 9 months<br>Patient Duration: maximum of 4 weeks	
Planned Interim Analyses	Not applicable	
STATISTICS<br>Primary Analysis Plan	For the primary endpoint, a two-sided paired Wilcoxon signed-rank test will be used to test the difference in urinary bladder SUV_{mean} between piflufolastat (^{18}F) and flotufolastat (^{18}F). This will be performed in patients who have received both radiopharmaceuticals and have both PET scans available.<br>Secondary and tertiary endpoints will be summarized descriptively, with 95% confidence intervals presented where applicable.<br>Safety data will be summarized descriptively.	
Rationale for Number of Patients	Approximately 60 patients will be screened for inclusion into the study to obtain a minimum of 52 evaluable patients (i.e., have evaluable PET scans for both piflufolastat (^{18}F) and flotufolastat (^{18}F) available for analysis).<br>A sample size of 52 evaluable patients achieves 80% power to detect a mean of paired differences of 10 in SUV_{mean}, with an estimated standard deviation of paired differences of 25 and with a significance level of 0.05, using a two-sided paired Wilcoxon signed-rank test, assuming that the actual distribution of paired differences is uniform.	

STUDY SCHEMA

Figure 1: Overall Study Design

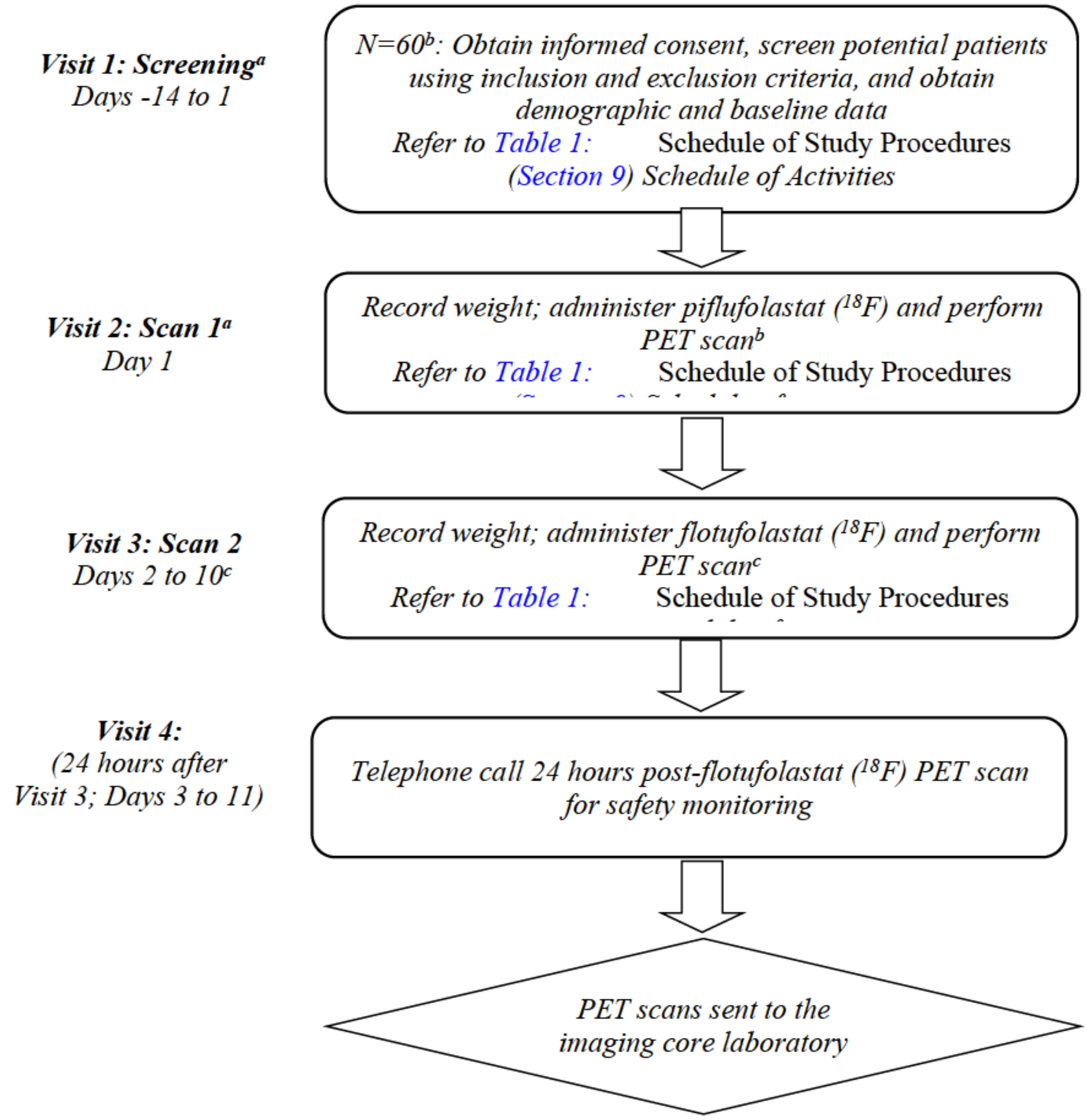

Abbreviations: ^{18}F=fluorine-18; PET=positron emission tomography.

[a] Visit 1 (screening) and Visit 2 (Scan 1) may be combined if the investigator decides to screen a patient and perform the piflufolastat (^{18}F) PET scan on the same day. In such cases, all assessments listed for Visit 1 (screening) should be performed **prior to** the piflufolastat (^{18}F) injection and PET scan. If Visit 1 (screening) and Visit 2 (Scan 1) are combined, the investigator should ensure ample time is made available for the patient to think about the study and ask questions before agreeing to participate (see Section 17.3 for details of informed consent requirements).

[b] Approximately 60 patients will be screened for inclusion into the study to obtain a minimum of 52 evaluable patients (i.e., have evaluable PET scans for both piflufolastat (^{18}F) and flotufolastat (^{18}F) available for analysis).

[c] The flotufolastat (^{18}F) PET scan should occur at least 24 hours later than, but within 10 calendar days of the piflufolastat (^{18}F) scan.

1 BACKGROUND

The investigational product (IP) in this Phase 4 study is flotufolastat (^{18}F) injection (POSLUMA®; Blue Earth Diagnostics Ltd, hereafter referred to as BED). This was approved by the United States (US) Food and Drug Administration (FDA) on 25 May 2023 as a radioactive diagnostic agent for positron emission tomography (PET) of prostate-specific membrane antigen (PSMA) positive lesions in men with prostate cancer:

- with suspected metastasis who are candidates for initial definitive therapy;
- with suspected recurrence based on elevated serum prostate-specific antigen (PSA) level.

POSLUMA is a ^{18}F-labeled PET ligand designed to target the extracellular epitope of the PSMA molecule and is administered as a single intravenous (IV) bolus microdose (i.e., the mass dose administered is less than 100 µg/patient). The molecular structure of the drug substance comprises a glutamate-urea PSMA binding motif, a peptide spacer and two potential binding sites to carry a radioactive tracer: (1) a -silicon fluorine acceptor (SiFA-) moiety, which covalently binds ^{18}F for PET imaging and (2) a chelator-metal complex (DOTAGAgallium-) that could alternatively be used for radiolabeling with a radiometal (Figure 4 [Section 8.3]). Having both a DOTAGA complex and a hydrocarbon-enhanced SiFA moiety in close proximity facilitates high PSMA targeting and internalization, which can result in an appropriate balance of tumor identification and uptake while minimizing a rapid and extensive occurrence of radioactivity in the bladder (Wurzer et al, 2020).

The comparator in this study, is also a US FDA -approved ^{18}F-labeled PSMA PET ligand, piflufolastat (^{18}F) injection, which is commercially available in the US under the trade name PYLARIFY® (Progenics Pharmaceuticals, Inc), and in the European Union (EU) under the trade name PYLCLARI® (Curium PET France). Despite demonstrating diagnostic performance in clinical trials and receiving FDA-approval, piflufolastat (^{18}F) may have limitations to its diagnostic use due to the level of urinary radioactivity demonstrated on PET scans (see Section 1.3 for further details). Consideration of the urinary tract is critical in the development of PSMA ligands as intense urinary retention may interfere with accurate evaluation of the prostate and adjacent area (Knorr et al, 2022).

During non-clinical and clinical development, both radiopharmaceuticals have been known by alternative names and may be referred to by these names in study reports and the published literature:

- **Flotufolastat (**^{18}F**) injection**: ^{18}F-rhPSMA-7.3, rhPSMA-7.3 (^{18}F), [^{18}F]rhPSMA-7.3, ^{18}F-flotufolastat, flotufolastat F 18 and F-18 flotufolastat PSMA;
- **Piflufolastat (**^{18}F**) injection**: ^{18}F-DCFPyL, [^{18}F]DCFPyL, piflufolastat F 18.

1.1 Overview of Non-clinical Studies

Consistent with the development of a microdose diagnostic radiopharmaceutical imaging agent, flotufolastat (^{18}F) is not designed to elicit pharmacological activity, and an *in vitro* secondary pharmacology screen in a panel of 44 potential targets confirmed no pharmacological activity associated with administration of the agent (Eurofins Cerep Study Number 100045872).

The toxicokinetics of flotufolastat in the rat indicated that exposure increased with increasing dose level from 0.1 to 10 mg/kg, and the increases in the maximum observed concentration, area under the concentration time curve (AUC) from hour 0 to the time of last measurable concentration (AUC_{0-t}), and AUC from hour 0 to 24 hours post-dose (AUC_{0-24}) values were dose proportional (Study Number 8391451). *In vitro* protein binding indicated moderate

binding of 75% and 82% for rat and human, respectively, with no concentration dependence (Study Number 8391453). *In vitro* studies indicated that flotufolastat was not metabolized by human cytochrome P450 (CYP) and uridine diphosphate glucuronyltransferase enzymes (Study Number 8391457). An *in vivo* absorption, distribution, metabolism, and excretion study in the rat indicated high plasma radioactivity concentrations (Study Number 8391458). Excretion was predominantly via the urine, with minor fecal elimination observed. *In vitro*, flotufolastat did not inhibit or induce human CYP isoforms and was not a substrate or inhibitor of human drug transporters, suggesting that drug interactions with flotufolastat are unlikely (Study Numbers 8391455, 8391454 and 8391456).

Non-clinical biodistribution and dosimetry data for flotufolastat (^{18}F) in mice demonstrated that retention in the urinary bladder was comparatively low (Knorr et al, 2022). Data demonstrated that the largest accumulation of radioactivity following flotufolastat (^{18}F) administration was in the kidney, spleen, lung, liver, and heart. Clearance from the blood and clearance to the urine was rapid for flotufolastat (^{18}F), but there was a relatively slow build-up of radioactivity in the kidney. Using a 3.5-hour and 1.0-hour bladder voiding interval, the total effective doses for humans extrapolated from the animal data were 21.7 and 12.8 microSievert (µSv)/megaBecquerel (MBq) for flotufolastat (^{18}F), respectively.

For further details of the flotufolastat (^{18}F) non-clinical development program refer to the flotufolastat (^{18}F) injection (Posluma®) Investigator's Brochure (IB).

1.2 Overview of Clinical Studies

Approval of flotufolastat (^{18}F) injection in the US was based on pivotal data from two Phase 3 diagnostic trials with flotufolastat (^{18}F) injection conducted in the US and EU (Studies BED-PSMA-301 [LIGHTHOUSE; subsequently published by Chapin, 2022; Kuo et al, 2024; Kuo et al, 2023; Surasi et al, 2023] and BED-PSMA-302 [SPOTLIGHT; subsequently published by Fleming, 2022; Kuo et al, 2022; Lowentritt, 2022; Helfand, 2023; Jani et al, 2023; Kuo et al, 2024]). Supportive data were provided from a Phase 1 study (BEDPSMA101; published by Malaspina et al, 2021; Tolvanen et al, 2021; Malaspina et al, 2022) conducted in Finland, and from real world use at the Technical University of Munich in Germany (Study BEDPSMA403, and publications by Rauscher et al, 2021; Kroenke et al, 2022; Langbein et al, 2022).

Phase 1 study: BED-PSMA-101

In the Phase 1 study (Study BED-PSMA-101), the biodistribution and internal radiation dosimetry of ^{18}F following IV administration of flotufolastat (^{18}F) was assessed in six healthy adult volunteers (Tolvanen et al, 2021). ^{18}F excretion into urine was 7.2% of the administered activity at 111 minutes post-injection. The highest mean initial uptake of ^{18}F was in the liver, heart content and kidneys. The most critical organs (i.e., those with the highest absorbed dose per unit of administered activity) were the adrenals, the kidneys, and the submandibular glands. The mean effective dose for men per unit of administered activity was 0.0138 milliSievert (mSv)/MBq using a 1 hour voiding interval and 0.0141 mSv/MBq using a 3.5 hour voiding interval. Pharmacokinetic analysis in both healthy volunteers and patients with prostate cancer showed that flotufolastat (^{18}F) did not undergo metabolism for up to 50 minutes post-injection. Efficacy data from nine patients with prostate cancer demonstrated that flotufolastat (^{18}F) uptake kinetics in lesions and relevant reference tissues was dominated by irreversible components. Optimal visual detection of flotufolastat (^{18}F) PET for the assessment of primary prostate tumors and metastatic lesions occurred between 60- and 90-minutes post-injection. The optimal time to commence scanning was therefore 60 minutes post-injection (Malaspina et al, 2021). In the patients studied, the radioactivity in the bladder did not impact assessment of the prostate tumor or pelvic lymph nodes (PLNs).

Phase 3 studies: BED-PSMA-301 and BED-PSMA-302

In the prospective, Phase 3, multi-center, single-arm, single dose marketing authorization studies, the safety and diagnostic performance of flotufolastat (^{18}F) were assessed in men with newly diagnosed unfavorable intermediate risk, high risk or very high risk prostate cancer (Study BED-PSMA-301 [LIGHTHOUSE]) and in men with biochemical recurrence (BCR) of prostate cancer based on elevated PSA levels (Study BED-PSMA-302 [SPOTLIGHT]). The totality of data from these studies demonstrated that flotufolastat (^{18}F) was well tolerated by patients and exhibited a favorable benefit to risk ratio for the detection of metastatic prostate cancer using PET imaging, including the identification of previously unrecognized N1 and M1 disease in newly diagnosed patients and localization of disease in patients with BCR of prostate cancer.

For further details of the flotufolastat (^{18}F) clinical program refer to the flotufolastat (^{18}F) injection (Posluma®) IB, approved POSLUMA United States Prescribing Information (USPI) and the cited published literature.

1.3 Urinary Radioactivity

Flotufolastat (^{18}F) was developed, in part, to leverage its potential for low bladder radioactivity due to its novel molecular structure. Preclinical data demonstrated that flotufolastat (^{18}F) retention in the urinary bladder is comparatively low (Knorr et al, 2022) and Phase 1 clinical data demonstrated that it has lower average urinary excretion compared to other renally cleared radiopharmaceuticals used for PSMA PET (Tolvanen et al, 2021). Because of this, flotufolastat (^{18}F) has the potential for improved image evaluation in the prostate and periureteric regions (Kuo et al, 2024). Specifically, based on the healthy volunteer cohort from BED-sponsored Study BED-PSMA-101, flotufolastat (^{18}F) in the urine was measured to be 7.2% (range: 4.4%, 9.0%), which is less than the average urinary excretion reported for other FDA-approved radiopharmaceuticals for PSMA PET (piflufolastat (^{18}F), as well as gallium (^{68}Ga) gozetotide; 11% each) in the first 2 hours (Tolvanen et al, 2021). Additionally, retrospective data from routine clinical use demonstrated good distinction between the primary tumor and background bladder radioactivity in patients with primary prostate cancer undergoing N-staging (Langbein et al, 2022).

To evaluate the impact of urinary radioactivity on flotufolastat (^{18}F) PET/computed tomography (CT) image interpretation and disease assessment, *post hoc* analysis of the flotufolastat (^{18}F) PET scans from the two BED-sponsored prospective Phase 3 studies (Study BED-PSMA-301 [LIGHTHOUSE] and Study BED-PSMA-302 [SPOTLIGHT]) have been performed (Kuo et al, 2024). For this analysis, 718 flotufolastat (^{18}F) PET scans, (352 from Study BED-PSMA-301 and 366 from Study BED-PSMA-302), were evaluated by three board-certified nuclear medicine readers trained to read flotufolastat (^{18}F) scans. For qualitative assessments, all three readers qualitatively assessed the impact of any urinary radioactivity in the bladder on image interpretation using a 3-point scale (0=no/minimal visible urinary radioactivity, 1=urinary radioactivity visible but distinction between urine and disease possible, and 2=assessment inhibited by urinary radioactivity), and the presence/absence of ureteric radioactivity and halo artifacts. Quantitative assessments (maximum standardized uptake value [SUV_{max}] and mean standardized uptake value [SUV_{mean}]) of bladder radioactivity were also performed, by placing a circular region of interest over the maximum diameter of the bladder radioactivity in the transverse plane.

Of the 718 eligible scans, 712 were evaluable for bladder radioactivity (348 with newly diagnosed prostate cancer; 364 with BCR of prostate cancer). Six scans were excluded from the evaluation due to cystectomy, renal failure, and urinary catheter in situ (two scans each). The median SUV_{max} in the bladder was 17.1 (interquartile range: 9.2, 28.3) and median SUV_{mean} was 12.5 (interquartile range: 7.0, 19.3; Figure 2).

Figure 2: Quantitative Assessment of Bladder Radioactivity: SUV_{max} and SUV_{mean}

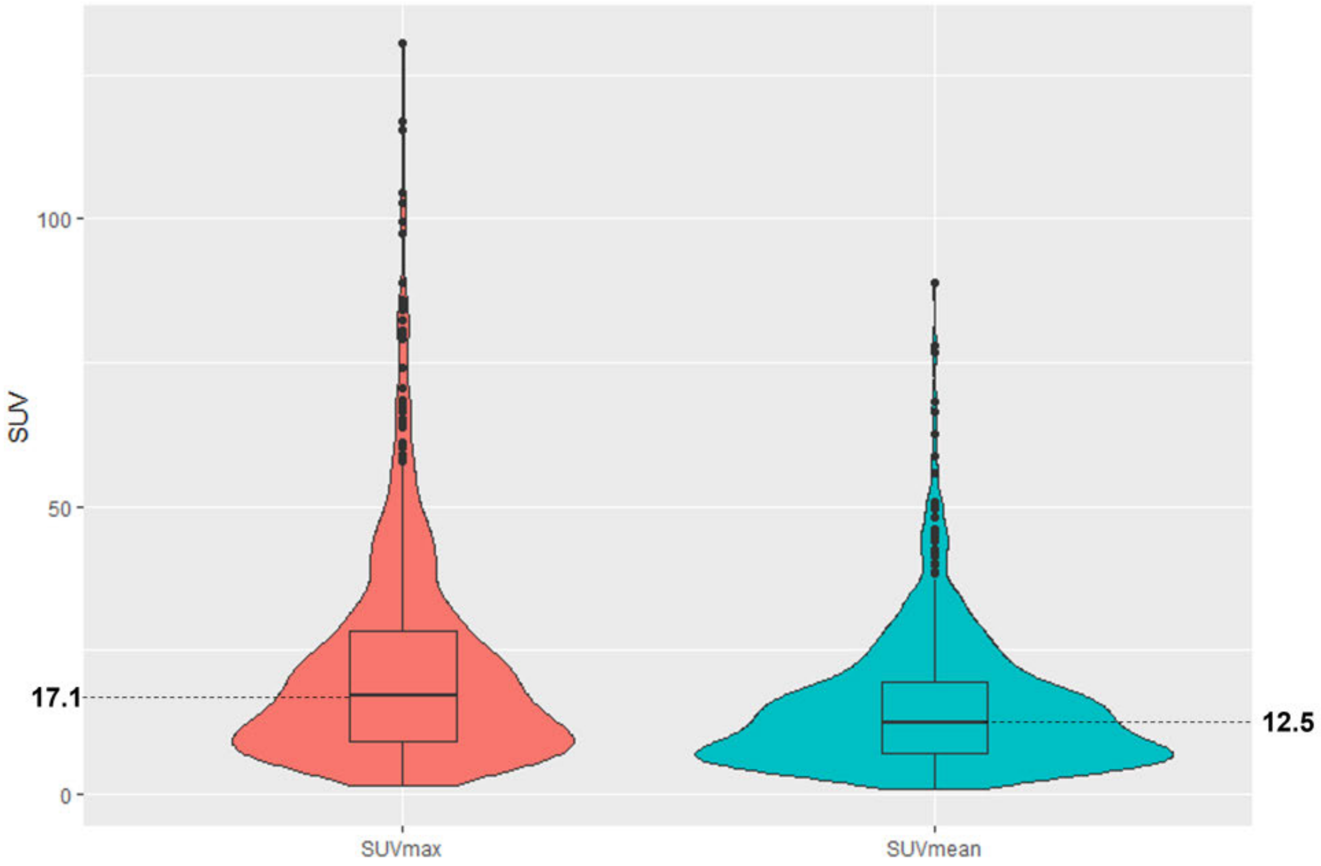

Abbreviations: SUV=standardized uptake value; SUV_{max}=maximum standardized uptake value; SUV_{mean}=mean standardized uptake value.
Source: Kuo et al, 2024

Furthermore, qualitative data demonstrated that in 682/712 (96%) patients by majority read, it was possible to distinguish between urinary radioactivity and disease uptake (i.e., rated 0 or 1). In the minority of patients (24/712 [3.4%]) in which urinary radioactivity did impact assessment (score=2), the median bladder SUV_{mean} was higher (20.5) than those scored 0 (3.8) or 1 (14.0). Ureteric radioactivity was absent in 401/712 (56%) patients (majority read). Halo artifacts around the bladder occurred in only 2/712 (0.3%) patients by majority read.

Thus, these data demonstrate that the urinary radioactivity of flotufolastat (^{18}F) was relatively low and did not influence disease assessment for the majority of patients. Moreover, while the study was not designed as a head-to-head comparison, the median bladder standardized uptake values (SUVs) of 17.1 (SUV_{max}) and 12.5 (SUV_{mean}) are lower than values reported in the literature for ^{18}F-DCFPyL (piflufolastat- (^{18}F): 61.7 to 79.3 [SUV_{max}]; Giesel et al, 2018; Donswijk et al, 2022) and other renally cleared PSMA PET ligands (^{68}Ga-PSMA-11: 43.5 to 68.4 [SUV_{max}] and 25.4 to 45.8 [SUV_{mean}]; Donswijk et al, 2022; Kuten et al, 2020; Uprimny et al, 2021; see Figure 3). Furthermore, halo artifacts were extremely rare with flotufolastat (^{18}F) PET, occurring in 0.3% of patients (majority read; Kuo et al, 2024). These are more likely to occur with higher kidney and bladder accumulation and have been reported to occur around the bladder and kidneys with ^{18}F-DCFPyL (Wondergem et al, 2019), as well as with other renally cleared radiopharmaceuticals (^{68}Ga-PSMA; Afshar-Oromieh et al, 2014; Rauscher et al, 2016; Heußer et al, 2017).

Figure 3: Quantitative Analysis of SUV_{max} and SUV_{mean} for Flotufolastat (^{18}F) Compared to Other Renally Cleared PSMA PET Ligands

Note: Data are from individual studies and do not represent a head-to-head comparison.

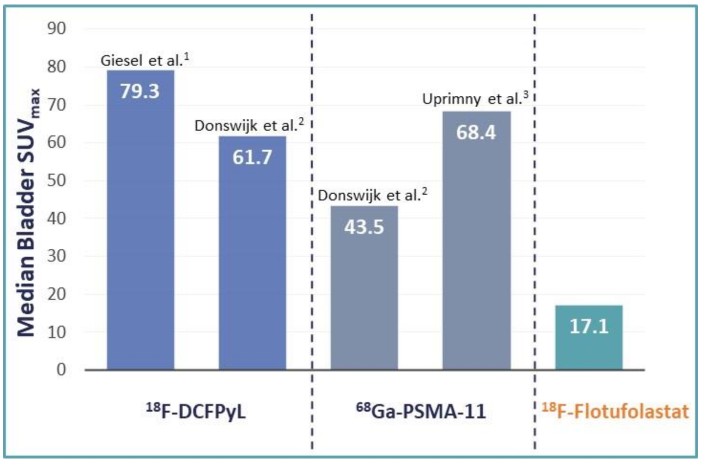

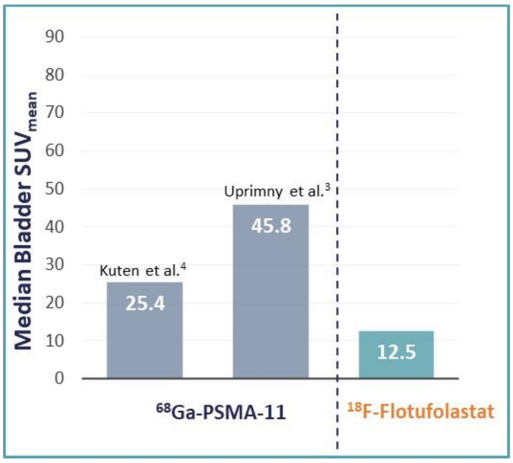

Abbreviations: ^{18}F=fluorine-18; ^{68}Ga=gallium-68; CT=computed tomography; DCFPyL=piflufolastat; PET=positron emission tomography; PSMA=prostate-specific membrane antigen; SUV_{max}=maximum standardized uptake value; SUV_{mean}=mean standardized uptake value; VOI=volume of interest.

1. Giesel et al, 2018 (n=12): The normal bladder was evaluated with a 2 cm sphere placed inside the organ parenchyma.
2. Donswijk et al, 2022 (n=51 [^{68}Ga-PSMA-11]; n=51 [^{18}F-DCFPyL]): By manually placing a VOI in the bladder over the hottest region, the SUV_{max} bladder corrected for body weight of the urinary radioactivity of ^{68}Ga-PSMA-11 and ^{18}FDCFPyLwas determined.
3. Uprimny et al, 2021 (n=50): VOIs were drawn automatically with a manually adapted isocontour threshold centered on the urinary bladder.
4. Kuten et al, 2020 (n=16): SUV_{mean} in the urinary bladder was automatically calculated from VOIs that were segmented automatically with a manually adapted isocontour threshold based on physiologic urine uptake and verified using anatomic CT data.

2 STUDY RATIONALE

PSMA PET has become a mainstay for the diagnostic imaging of both primary and BCR prostate cancer (Kuo et al, 2024). The kidneys and the urinary tract are critical organs in the development of PSMA ligands as intense urinary radioactivity may interfere with accurate evaluation of the prostate and adjacent area (Knorr et al, 2022). The interpretation of PET images can be influenced by the normal biodistribution of PSMA radiopharmaceuticals, particularly in the prostate/prostate bed and PLNs, where excreted urine can visually conceal the anatomical areas under assessment or appear visually indistinguishable from disease. Several approaches to minimize the impact of bladder radioactivity on image interpretation have been investigated, including early/late imaging and/or the co-administration of diuretics. However, results have varied and to ensure patient comfort and optimal integration into an existing workflow, a scanning protocol would ideally not require either of these approaches (Kuo et al, 2024).

Flotufolastat (^{18}F) was developed, in part, to leverage its potential for low bladder radioactivity due to its novel molecular structure. As described in Section 1.3, data from the BED-sponsored Phase 1 and Phase 3 flotufolastat (^{18}F) injection FDA marketing authorization studies demonstrate relatively low urinary radioactivity, on average, for flotufolastat (^{18}F) compared to those published for other approved renally cleared radiopharmaceuticals used for PSMA PET, including piflufolastat (^{18}F) (Giesel et al, 2018; Kuten et al, 2020; Uprimny et al, 2021; Donswijk et al, 2022), as well as a very low occurrence of halo artifacts (Kuo et al, 2024). This may represent a clinically meaningful difference for flotufolastat (^{18}F) given the challenge of delineating local recurrences (lesions within and adjacent to the prostate bed) of prostate cancer at low PSA values. However, the post-hoc analysis of the flotufolastat (^{18}F) PET scans from the two BED sponsored- prospective Phase 3 studies was not designed as a head-to-head comparison with other radiopharmaceuticals for PSMA PET, and thus, comparisons of the median SUVs across different studies, as described in Section 1.3, do not allow conclusions to be drawn.

This Phase 4 study is designed to be a direct head-to-head study of urinary radioactivity for flotufolastat (^{18}F) compared to piflufolastat (^{18}F) in patients with low PSA (≤0.5 ng/mL) BCR of prostate cancer following RP, in order to further provide valuable information on this biodistribution topic. In addition, the secondary and tertiary objectives of the study will provide supportive data for the two radiopharmaceuticals, with respect to detection rates and other organ biodistribution metrics in this population of patients with low PSA BCR of prostate cancer.

2.1 Risk-Benefit Assessment

Patients recruited into this study will be men with low PSA (≤0.5 ng/mL) BCR of prostate cancer following radical prostatectomy (RP) who are already scheduled to receive a (^{18}F) PET scan as part of routine clinical care.

In order to compare urinary radioactivity SUVs following piflufolastat (^{18}F) and flotufolastat (^{18}F) PET, patients will undergo two PSMA PET scans as part of this Phase 4 study: piflufolastat (^{18}F) (Scan 1) and flotufolastat (^{18}F) (Scan 2). Both radiopharmaceuticals used in this study have been approved by the US FDA for use in the intended patient population and, thus, have a favorable benefit to risk ratio when used in accordance with their respective approved USPIs.

The first scan (piflufolastat (^{18}F)) will have been scheduled by the treating physician as part of routine clinical care prior to the patient entering this study, and the patient will receive this scan irrespective of their participation in this study. The second scan (flotufolastat (^{18}F)) will only

be performed if the patient enters this study and may provide additional direct benefit to the patient (see Section 2.1.1) or may be associated with additional risks (see Section 2.1.2).

2.1.1 *Benefits*

As described in Section 2, the relatively low bladder radioactivity for flotufolastat (^{18}F), compared to those published for other approved radiopharmaceuticals for PSMA PET, may represent a clinically meaningful difference for flotufolastat (^{18}F) given the challenge of delineating local recurrences (lesions within and adjacent to the prostate bed) of prostate cancer at low PSA values.

The flotufolastat (^{18}F) PET scan may provide further clinical information regarding the patient's disease status that may not have been appreciated by routine clinical testing or by the piflufolastat (^{18}F) PET scan. If such information arises through interpretation of the PET scan by site readers, the findings should be reported back to the responsible clinician to help direct the patient's further management by caregivers at the site. This may provide a direct benefit to the patient.

2.1.2 *Risks*

Risks and mitigations associated with piflufolastat (^{18}F) and flotufolastat (^{18}F) PET are detailed in Section 5 of the respective approved USPIs and include the risks of image misinterpretation and radiation exposure for both radiopharmaceuticals.

As described in Section 2.1, patients will receive an (^{18}F) PET scan as part of their routine clinical care, irrespective of their participation in this study, but the flotufolastat (^{18}F) scan will only be performed if the patient enters this study. This additional scan may be associated with additional risks, as discussed below.

The additional flotufolastat (^{18}F) PET scan will contribute to a patient's overall long-term cumulative radiation exposure. Long-term cumulative radiation exposure is associated with an increased risk for cancer (POSLUMA USPI, Section 5.2 and PYLARIFY USPI, Section 5.3). Patients should be advised to hydrate before and after administration and to void frequently after administration, and safe handling should be ensured to minimize radiation exposure to the patient and health care providers (POSLUMA USPI, Section 5.2 and PYLARIFY USPI, Section 5.3). The effective radiation dose resulting from the administration of the recommended radioactivity of 296 MBq of flotufolastat (^{18}F) is 4.1 mSv (POSLUMA USPI, Section 2.6).

In the current study, the maximum effective dose due to the CT transmission scan on a PET/CT scanner will vary from site-to-site, but as a guide a dose of 7 mSv would be expected. The effective dose due to the CT acquisition will be in accordance with ALARA (As Low As Reasonably Achievable) principles. The estimated total dose is <12 mSv (PET [4.1 mSv] and CT transmission scan [7 mSv]) is in line with other common nuclear medicine procedures.

In the marketing authorization studies of flotufolastat (^{18}F), safety was evaluated in 747 patients with prostate cancer, all of whom received a single dose of flotufolastat (^{18}F), with a mean±standard deviation administered radioactivity of 307±23 MBq (8.3±0.6 milliCurie [mCi]). Flotufolastat (^{18}F) was well tolerated, with only the following adverse reactions reported in ≥0.4% of patients: diarrhea (five [0.7%] patients), blood pressure increase (four [0.5%] patients) and injection site pain (three [0.4%] patients). See Section 6.1 of the POSLUMA USPI for further details.

3 STUDY OBJECTIVES

3.1 Primary Objective

The primary objective of the study is to compare urinary bladder radioactivity observed on piflufolastat (^{18}F) PET and flotufolastat (^{18}F) PET.

3.2 Secondary Objectives

The secondary objectives of the study are to assess the following for piflufolastat (^{18}F) PET and flotufolastat (^{18}F) PET:

- Detection rates;
- Detection rates stratified by PSA level;
- Prostate bed detection rates: local recurrences by subregion;
- PLN detection rates.

3.3 Tertiary Objectives

The tertiary objectives of the study are to assess the following for piflufolastat (^{18}F) PET and flotufolastat (^{18}F) PET:

- Ureteric radioactivity;
- Bladder radioactivity;
- Physiological radioactivity;
- Extra-pelvic detection rates;
- Qualitative analysis of urinary radioactivity interference with image assessment.

3.4 Safety Objectives

Safety objectives will be to assess treatment-emergent adverse events (TEAEs) after each PSMA PET scan.

4 STUDY DESIGN

4.1 Study Overview

This is a multi-center, prospective intra-patient comparator study of urinary radioactivity SUV following both piflufolastat (^{18}F) and flotufolastat (^{18}F) PET in patients with low PSA (≤0.5 ng/mL) BCR of prostate cancer following RP. Approximately 60 patients will be screened for inclusion into the study to obtain a minimum of 52 evaluable patients (i.e., have evaluable PET scans for both piflufolastat (^{18}F) and flotufolastat (^{18}F) available for analysis). Each patient will be administered a single dose of piflufolastat (^{18}F) on Day 1, followed by a PET scan per standard institutional practice and in line with the PYLARIFY USPI. At least 24 hours after the piflufolastat (^{18}F) scan, but within 10 calendar days, all patients will be administered a single dose of flotufolastat (^{18}F) followed by a PET scan. Each patient will receive both radiopharmaceuticals and, thus, serve as their own control.

For the primary endpoint analysis, volumes of interest (VOIs) will be placed over the urinary bladder, and radioactivity (SUV metrics) will be assessed following administration of each radiopharmaceutical.

For secondary and tertiary endpoints, both the piflufolastat (^{18}F) and flotufolastat (^{18}F) PET scans will be read by two independent nuclear medicine physicians or nuclear radiologists who are blinded to the patient's clinical history, but have been trained to read scans for each respective radiopharmaceutical using product-specific training and in line with the FDA approved USPI for the respective radiopharmaceutical. Any image interpretation disagreements will be resolved by a third independent adjudicator.

Patients will be screened within 14 days of Visit 1 (Days -14 to 1) and screening data will be reviewed to determine patient eligibility. Patients who meet all of the inclusion criteria and none of the exclusion criteria will be entered into the study.

The following regimens will be used:

- Scan 1 (Visit 2; Day 1)[1]: Piflufolastat (^{18}F) injection and PET scan;
- Scan 2 (Visit 3; Days 2 to 10): Flotufolastat (^{18}F) injection and PET scan.

Patients will subsequently receive a telephone call 24 hours after Scan 2 (Visit 4; Days 3 to 11) for safety monitoring.

The total duration of participation for each patient will be a maximum of 4 weeks. The total duration of the study is expected to be 6 to 9 months.

[1] Visit 1 (screening) and Visit 2 (Scan 1) may be combined if the investigator decides to screen a patient and perform the piflufolastat (^{18}F) PET scan on the same day. In such cases, all assessments listed for Visit 1 (screening) should be performed **prior to** the piflufolastat (^{18}F) injection and PET scan.

5 CRITERIA FOR EVALUATION

5.1 Primary Endpoint

The primary endpoint will be assessed centrally as the difference in urinary bladder SUV_{mean} between piflufolastat (^{18}F) and flotufolastat (^{18}F).

The SUV is a measure of the relative radioactivity uptake in a region of interest and will permit a direct head-to-head comparison of urinary bladder radioactivity between the two radiopharmaceuticals (piflufolastat (^{18}F) and flotufolastat (^{18}F)).

5.2 Secondary Endpoints

The secondary endpoints will be an estimate of the following for each radiopharmaceutical following a central read:

- Patient-level detection rate;
- Patient-level detection rate by baseline PSA categories;
- Detection rate for local recurrences in prostate bed for the following subregions:
    - vesicourethral anastomosis lesions;
    - retrovesical lesions;
    - remnant seminal vesicles/lateral surgical margin lesions;
- Detection rate for PLN lesions.

These secondary endpoints will provide supportive data for the two radiopharmaceuticals (piflufolastat (^{18}F) and flotufolastat (^{18}F)) in terms of detection rates.

5.3 Tertiary Endpoints

The tertiary endpoints will be an estimate of the following for each radiopharmaceutical:

- Central reader qualitative assessment of the presence or absence of ureteric radioactivity;
- SUV_{max}, peak SUV (SUV_{peak}) and total bladder radioactivity;
- SUV_{mean}, SUV_{peak} for normal organs, including liver and spleen;
- Detection rate for distant metastatic lesions and in the following subregions:
    - bone lesions;
    - extra-pelvic lymph node lesions;
    - extra-pelvic soft tissue lesions;
- Qualitative 3-point scale by expert readers (as previously described by Kuo et al, 2024).

These tertiary endpoints will provide further supportive data for the two radiopharmaceuticals (piflufolastat (^{18}F) and flotufolastat (^{18}F)) in terms of biodistribution and detection rates for distant metastases.

5.4 Safety Evaluations

- Incidence of TEAEs after each PSMA PET scan.

6 PATIENT SELECTION

6.1 Study Population

Patients with low PSA (≤0.5 ng/mL) BCR of prostate cancer following RP who are scheduled to receive a PSMA (^{18}F) PET scan, and who meet all of the inclusion criteria and none of the exclusion criteria, will be eligible for participation in this study.

6.2 Inclusion Criteria

1. Male ≥18 years of age at Visit 1 (Screening).
2. Documented history of localized adenocarcinoma of the prostate with prior curative intent treatment with RP , experiencing BCR of prostate cancer.
   a. At least 6 months must have elapsed after RP.
3. Low PSA BCR defined as PSA ≤0.5 ng/mL.
4. Scheduled by their treating physician to receive a PSMA (^{18}F) PET scan.
5. Written informed consent obtained from the patient and ability for the patient to comply with the requirements of the study.

6.3 Exclusion Criteria

1. Patients with any medical condition or circumstance (including receiving an IP) that the investigator believes may compromise the data collected or lead to a failure to fulfill the study requirements.
2. Patients who are planned to have an x-ray contrast agent or any other PET radiotracer within 24 hours prior to either PSMA PET scan.
3. Patients who have already received a piflufolastat (^{18}F) PET scan for BCR within 8 weeks prior to providing informed consent for this study.
4. Patients participating in an interventional clinical trial within 30 days and having received an IP within five biological half-lives prior to either of the PSMA PET scans in this study.
5. Patients with known hypersensitivity to the active substance or to any of the excipients of piflufolastat (^{18}F) or flotufolastat (^{18}F).
6. Patients who have previously undergone a cystectomy or have renal failure or have other conditions that may significantly affect urinary output, as judged by the investigator.
7. Patients who have received salvage therapy for the current episode of BCR.

7 CONCOMITANT MEDICATIONS

All patients should be maintained on the same medications from screening through to the post--flotufolastat (^{18}F) safety monitoring telephone call, as medically feasible.

7.1 Allowed Medications and Treatments

Standard therapy for prostate cancer is allowed except for treatments noted in the exclusion criteria described above in Section 6.3 and as noted in the prohibited medications section below (Section 7.2).

7.2 Prohibited Medications and Treatments

The following medications are prohibited during the study and administration will be considered a protocol violation:

- X-ray contrast agent within 24 hours prior to the piflufolastat (^{18}F) or flotufolastat (^{18}F) PET scans;
- Any other PET radiotracer within 24 hours prior to the piflufolastat (^{18}F) or flotufolastat (^{18}F) PET scans;
- Concurrent use of diuretics (e.g., Lasix [furosemide]) or urinary catheterization (for the purpose of improving PET images) are not permitted for either PSMA PET scan.

8 STUDY TREATMENTS

8.1 Method of Assigning Patients to Treatment Groups

All patients will receive both radiopharmaceuticals and PET scans and, thus, serve as their own control: piflufolastat (^{18}F) first (Day 1), followed at least 24 hours later, but within 10 calendar days, by flotufolastat (^{18}F).

8.2 Blinding

The local sites, including the investigator, will not be blinded as each patient will receive piflufolastat (^{18}F) first (Day 1), followed by flotufolastat (^{18}F). To allow use of the appropriate agent specific interpretation criteria, central readers will be unblinded to the radiopharmaceutical but will be blinded to all other clinical data.

8.3 Formulation of the Investigational Product

Flotufolastat (^{18}F) is an ^{18}F-labeled PSMA radiopharmaceutical diagnostic imaging agent. The active ingredient is flotufolastat (^{18}F) gallium. The molecular structure includes a DOTAGA complex with a nonradioactive, naturally occurring gallium ion. Radioactive ^{18}F is covalently bound to a SiFA moiety.

Chemically, flotufolastat (^{18}F) gallium is gallate(6-), [(*4S,8S,13R,27R,30R,35S*)-35-[4,10-bis[(carboxy-k*O*)methyl]-7-(carboxymethyl)-1,4,7,10-tetraazacyclododec-1-yl-kN^1,kN^4,kN^7,kN^{10}]-30-[[[4-[bis(1,1-dimethylethyl)fluoro-^{18}F-silyl]benzoyl]amino]methyl]-1,36-dihydroxy-1,6,11,18,21,29,32,36-octaoxo-5,7,12,17,22,28,31-heptaazahexatriacontane-4,8,13,27-tetracarboxylato(9-)]-, hydrogen (1:6).

The molecular weight is 1537.3 g/mol and the structural formula is shown in Figure 4.

Figure 4: Structural Formula of Flotufolastat (^{18}F) Injection

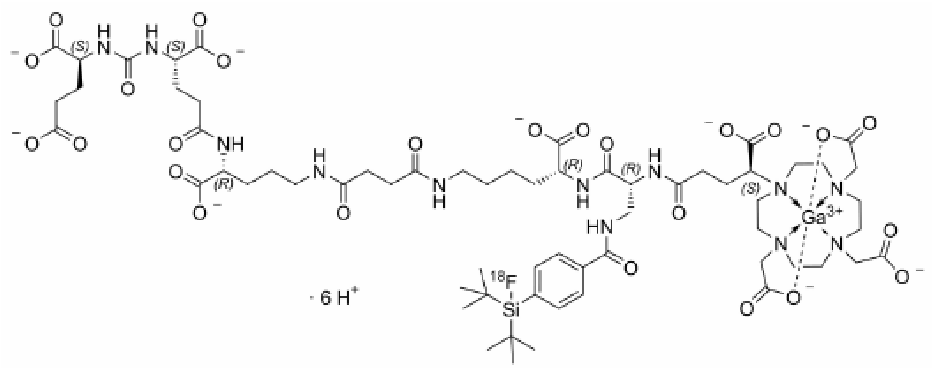

Flotufolastat (^{18}F) injection is a sterile, non-pyrogenic, clear, colorless, and isotonic solution. Each mL contains up to 20 µg of flotufolastat gallium, up to 5,846 MBq (158 mCi) as flotufolastat (^{18}F) gallium at end of synthesis, and the following inactive ingredients: not more than 10% (v/v) alcohol, 1.9 mg anhydrous citric acid, 7.2 mg sodium chloride, and 0.75 mg sodium hydroxide to adjust pH between 4 and 6. Flotufolastat (^{18}F) injection contains no preservative.

See POSLUMA USPI for further details.

8.4 Formulation of Comparator

Piflufolastat (^{18}F) injection contains ^{18}F, radiolabeled PSMA inhibitor imaging agent. Chemically piflufolastat (^{18}F) is 2-(3-{1-carboxy-5-[(6-[18F]fluoro-pyridine-3-carbonyl)-amino]-pentyl}ureido)-pentanedioic acid. The molecular weight is 441.4 and the structural formula is shown in Figure 5.

Figure 5: Structural Formula of Piflufolastat (^{18}F) Injection

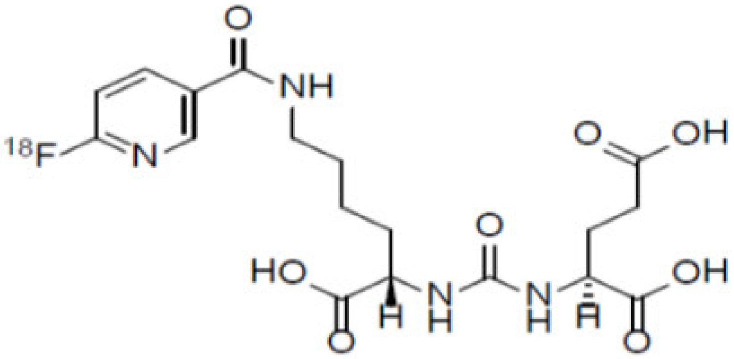

The chiral purity of the unlabeled piflufolastat (^{18}F) precursor is greater than 99% (S,S). Piflufolastat (^{18}F) injection is a sterile, non-pyrogenic, clear, colorless solution for IV injection. Each mL contains 37 to 2,960 MBq (1 to 80 mCi) piflufolastat (^{18}F) with ≤0.01 µg/mCi of piflufolastat at calibration time and date, and ≤78.9 mg ethanol in 0.9% sodium chloride injection United States Pharmacopeia (USP). The pH of the solution is 4.5 to 7.0. Piflufolastat (^{18}F) injection has a radiochemical purity of at least 95% up to 10 hours following end of synthesis, and specific activity of at least 1,000 mCi/µmol at the time of administration.

See PYLARIFY USPI for further details.

8.5 Packaging and Labeling

8.5.1 *Flotufolastat (^{18}F) Injection*

Flotufolastat (^{18}F) injection will be supplied in a patient ready, single-use syringe. The syringe label will include the following information: Rx number, product expiry date/time, total product volume (dispensed), radioactive concentration and/or total radioactivity (activity in mCi and/or MBq), calibration date and time, and an investigational use statement.

8.5.2 *Piflufolastat (^{18}F) Injection*

Piflufolastat (^{18}F) injection will be supplied and labeled as per commercially available product.

8.6 Radiopharmaceutical Supply at the Site

Sites will be provided with instructions for ordering flotufolastat (^{18}F) injection for use in the study. The specific date and time for a patient to be scanned needs to be included when placing the order. Flotufolastat (^{18}F) injection will be delivered from the radiopharmacy to the imaging site by courier. Each syringe is supplied in a container providing appropriate radiation shielding. The site must keep records of all shipments of flotufolastat (^{18}F) injection received, dispensing and disposal/destruction performed on-site as is appropriate to each facility.

Piflufolastat (^{18}F) injection will be obtained from commercial supply as per standard institutional practice.

8.7 Dosage/Dosage Regimen

Each patient will receive piflufolastat (^{18}F) first (Day 1; Visit 2), followed at least 24 hours later, but within 10 calendar days, by flotufolastat (^{18}F). Both radiopharmaceuticals will be administered as single doses according to their respective approved USPIs.

For the IP (flotufolastat (^{18}F) injection), the recommended amount of radioactivity to be administered is 296±20% MBq (8±20% mCi) administered as an IV bolus injection.

See PYLARIFY USPI and POSLUMA USPI for further details.

8.8 Dispensing

Both piflufolastat (^{18}F) and flotufolastat (^{18}F) injections should only be used by authorized persons who are qualified by specific training and are experienced in the safe use and handling of radionuclides, and whose experience and training have been approved by the appropriate government agency authorized to license the use of radionuclides.

Dispensing should be according to the respective approved USPIs for piflufolastat (^{18}F) and flotufolastat (^{18}F) (PYLARIFY USPI and POSLUMA USPI).

8.9 Administration Instructions

Both piflufolastat (^{18}F) and flotufolastat (^{18}F) will be administered as single doses according to their respective approved USPIs. Full preparation and administration instructions for each radiopharmaceutical, including post-administration details are provided in the respective USPIs. For both radiopharmaceuticals:

- Use aseptic technique and radiation shielding when withdrawing/preparing and administering;
- Visually inspect before administration. Do not use the radiopharmaceuticals if the solution contains particulate matter or is discolored (both radiopharmaceuticals are clear, colorless solutions);
- Calculate the necessary volume to administer based on calibration time and required dose. Both radiopharmaceuticals may be diluted with 0.9% Sodium Chloride Injection, USP;
- Assay the dose in a suitable dose calibrator before administration;
- Follow the injection with an IV flush of 0.9% Sodium Chloride Injection USP to ensure full delivery of the dose;
- Dispose of any unused radiopharmaceutical in compliance with applicable regulations.

See PYLARIFY USPI and POSLUMA USPI for further details.

8.10 Storage

Both piflufolastat (^{18}F) and flotufolastat (^{18}F) injections should be stored according to their respective approved USPIs.

Store both radiopharmaceuticals at controlled room temperature (USP) 20°C to 25°C (68°F to 77°F) in the original containers with radiation shielding. The expiration date and time are provided on the container labels. Use both radiopharmaceuticals within 10 hours from the time of the end of synthesis.

Dispose of any unused radiopharmaceutical in compliance with applicable regulations.

See PYLARIFY USPI and POSLUMA USPI for further details.

8.11 Investigational Product Accountability

An accurate and current accounting of the dispensing and disposal/return of flotufolastat (^{18}F) for each patient will be recorded on the Investigational Drug Accountability Record. The study monitor will verify this document throughout the course of the study.

8.12 Measures of Treatment Compliance

For piflufolastat (^{18}F), the administered radioactivity will be obtained for each patient from their medical records and recorded in the electronic case report form (eCRF).

For flotufolastat (^{18}F), the batch number and radioactivity per administration (determined by the radioactivity in the injection device before and after administration, with measurement date and time) will be recorded in each patient's eCRF/source document.

8.13 Primary Study Completion and Participant Completion Date

The end of patient participation in this study is defined as when the last patient has completed Visit 4 (post-procedural safety monitoring telephone call 24 hours after the second PSMA PET scan [Visit 3]).

The primary study completion date is defined as when the last blinded image evaluations have been completed.

9 STUDY PROCEDURES AND GUIDELINES

A Schedule of Events representing the required testing procedures to be performed for the duration of the study is given below in Table 1:

Table 1: Schedule of Study Procedures

	Visit 1[a] Screening Days -14 to 1	Visit 2[a] Scan 1[b] Day 1	Visit 3 Scan 2[b] Days 2 to 10[c]	Visit 4 24 Hours After Scan 2 Days 3 to 11[c] (Telephone)
Informed consent[a]	X			
Eligibility criteria	X			
Demographics	X			
Prostate cancer history (diagnosis, prior therapies)	X			
General medical history	X			
PSA sample (within 14 days of Visit 2)[d]	X			
Height	X			
Weight	X	X[e]	X[e]	
Administration of piflufolastat (^{18}F) injection and PET scan		X[f]		
Administration of flotufolastat (^{18}F) injection and PET scan			X[f]	
Post-flotufolastat (^{18}F) 24-hour telephone call				X
AEs[g]	X			
Concomitant medications	X			

Abbreviations: ^{18}F=fluorine-18; AE=adverse event; PET=positron emission tomography; PSA=prostate-specific antigen; TEAE=treatment-emergent adverse event.

[a] Visit 1 (screening) and Visit 2 (Scan 1) may be combined if the investigator decides to screen a patient and perform the piflufolastat (^{18}F) PET scan on the same day. In such cases, all assessments listed for Visit 1 (screening) should be performed prior to the piflufolastat (^{18}F) injection and PET scan. If Visit 1 (screening) and Visit 2 (Scan 1) are combined, the investigator should ensure ample time is made available for the patient to think about the study and ask questions before agreeing to participate (see Section 17.3 for details of informed consent requirements).

[b] All patients will receive the PSMA PET scans in the following order: piflufolastat (^{18}F) (Scan 1) and flotufolastat (^{18}F) (Scan 2).

[c] The flotufolastat (^{18}F) PET scan should occur at least 24 hours later than, but within 10 calendar days of the piflufolastat (^{18}F) scan.

[d] If a routine PSA sample is not collected within 14 days of Visit 2, then investigator can collect a separate PSA sample within 14 days of Visit 2.

[e] At Visits 2 and 3, weight should be recorded before radiopharmaceutical administration.

[f] For full details of the PET scan see the Study Imaging Manual (also known as Technical Operations Manual).

[g] AEs will be monitored and recorded from the time of informed consent until the last study visit; however, given the piflufolastat (^{18}F) injection will be administered as part of routine clinical care, any TEAEs attributable to piflufolastat (^{18}F) **will not be** assigned causality (related/not related to piflufolastat (^{18}F); see Section 12 for details).

Prior to conducting any study-related activities, written informed consent (and assent, if applicable) and the Health Insurance Portability and Accountability Act of 1996 (HIPAA) authorization must be signed and dated by the patient (Section 17.3).

9.1 Clinical Assessments

9.1.1 Concomitant Medications

All concomitant medication will be documented at screening, at all visits and at early termination when applicable, with any changes in concomitant medication during the study recorded in the eCRF. The dose, route, unit frequency of administration, indication for administration and dates of medication will be captured.

9.1.2 Demographic and Baseline Characteristics

Demographic information (year of birth, ethnicity/race, height, weight, as permitted by local regulations) will be recorded at screening. Weight will also be recorded on the day of each PET scan (Visits 2 and 3) prior to administration of each radiopharmaceutical.

9.1.3 Medical History

Medical history, including concomitant disease record, information regarding underlying chronic diseases and family history of cancer will be recorded at screening. Prostate cancer history (diagnosis and prior therapies) and other clinically significant diagnoses that may affect study assessments (e.g., overactive bladder, urinary stasis), in the judgment of the investigator, will be recorded.

9.1.4 Imaging Studies

All patients will receive the PSMA PET scans in the following order: piflufolastat (^{18}F) (Scan 1; Visit 2) and flotufolastat (^{18}F) (Scan 2; Visit 3). Further details of the imaging acquisition protocol are provided in Section 11.

9.1.5 Physical Examination

Not applicable.

9.1.6 Vital Signs

Not applicable.

9.1.7 PSA Levels

Patients' PSA level will be collected at screening (within 14 days of Visit 2) according to site standard of care laboratory testing.

9.2 Clinical Laboratory Measurements

Not applicable.

10 EVALUATIONS BY VISIT

10.1 Visit 1 – Screening (Days -14 to 1)

1. Review the study with the patient and obtain written informed consent and HIPAA authorization.
2. Assign the patient a unique screening number.
3. Review eligibility criteria and patient suitability for enrollment.
4. Record demographic data, height and weight, medical history (general and prostate cancer history) and concomitant medications.
5. Collect PSA value according to site standard of care laboratory testing (within 14 days of Visit 2).
6. Commence AE monitoring.
7. Schedule patient for Visit 2 within 14 days of consent.

10.2 Visit 2 – Scan 1 (Day 1)

1. Record any changes to concomitant medications and any AEs since screening (Visit 1).
2. Record pre-piflufolastat (^{18}F) administration weight.
3. Administer piflufolastat (^{18}F) and perform PET scan. Results of the image interpretation by the local site reader should be recorded in the eCRF.
4. Schedule patient for Visit 3, which should be at least 24 hours after the piflufolastat (^{18}F) PET scan, but within 10 calendar days.

Note: Visit 1 (screening) and Visit 2 (Scan 1) may be combined if the investigator decides to screen a patient and perform the piflufolastat (^{18}F) PET scan on the same day. In such cases:

- **All assessments listed in Section 10.1 (Visit 1 [screening]) should be performed prior to the piflufolastat (^{18}F) injection and PET scan;**
- **The investigator should ensure ample time is made available for the patient to think about the study and ask questions before agreeing to participate (see Section 17.3 for details of informed consent requirements).**

10.3 Visit 3 – Scan 2 (Days 2 to 10)

Note: this visit should occur at least 24 hours later than, but within 10 calendar days of Scan 1.

1. Record any changes to concomitant medications and any AEs since Visit 2 (Day 1).
2. Record pre-flotufolastat (^{18}F) administration weight.
3. Administer flotufolastat (^{18}F) and perform PET scan. Results of the image interpretation by the local site reader should be recorded in the eCRF.
4. Schedule patient for a telephone call (Visit 4) on the day after the scan.

10.4 Visit 4 – 24 Hours After Scan 2 (Days 3 to 11; Telephone)

1. Perform post-flotufolastat (^{18}F) scan safety monitoring telephone call, including recording any AEs and changes to concomitant medications since Visit 3.

11 IMAGING PROTOCOL

A summary of the imaging protocol for piflufolastat (^{18}F) (Scan 1 [Visit 2]; as part of routine clinical care) and flotufolastat (^{18}F) (Scan 2 [Visit 3]) is provided below in Sections 11.1 to 11.5. Full details are in the Study Imaging Manual (aka Technical Operations Manual).

11.1 PET Scanner

A dedicated hybrid PET/CT is mandatory. The selected scanner must be approved by the imaging core laboratory (Invicro, LLC). For each patient, the same PET/CT scanner must be used for both the piflufolastat (^{18}F) PET scan (Scan 1 [Visit 2]) and the flotufolastat (^{18}F) PET scan (Scan 2 [Visit 3]). Full details are in the Study Imaging Manual (aka Technical Operations Manual).

11.2 Injection Administration

See Section 8.9 for a summary of the administration instructions for each radiopharmaceutical. Both radiopharmaceuticals will be administered as single doses according to their respective approved USPIs.

See Section 2.2 of the PYLARIFY USPI and POSLUMA USPI for further details.

11.3 PET/CT Acquisition

The piflufolastat (^{18}F) PET scan (Scan 1) will be performed on Day 1 (Visit 2) per standard institutional practice and in line with the PYLARIFY USPI. For each patient, the flotufolastat (^{18}F) scan acquisition technical parameters should be equivalent to the piflufolastat (^{18}F) PET scan acquisition protocol for that particular site.

- Concurrent use of diuretics (e.g., Lasix [furosemide]) or urinary catheterization (for the purpose of improving PET images) are not permitted for either PSMA PET scan (see Section 7.2).
- Patients should not have an x-ray contrast agent or any other PET radiotracer within 24 hours prior to either PSMA PET scan (see Section 7.2).
- Patients should void immediately prior to imaging.
- Position the patient supine with arms above the head.
- For piflufolastat (^{18}F), begin image acquisition 50 to 90 minutes post-injection.
- For flotufolastat (^{18}F), begin image acquisition 50 to 70 minutes post-injection.
- Axial scan length for both scans will be matched to the piflufolastat (^{18}F) scan length at site.
- Scan duration is 12 minutes to 40 minutes depending on the number of bed positions (typically six to eight) and acquisition time per bed position (typically 2 minutes to 5 minutes).
- A minimum time of 24 hours is required between the PSMA PET (i.e., piflufolastat (^{18}F) and flotufolastat (^{18}F)) scan acquisitions.

Full details of the image acquisition protocol are in the Study Imaging Manual (also known as Technical Operations Manual).

Patients should be instructed to drink water prior to administration of each radiopharmaceutical to ensure adequate hydration, and to continue drinking and voiding frequently for the first few hours following administration to reduce radiation exposure.

11.4 Image Transfer

PET scan data requires central assessment and therefore images should be submitted as soon as the scan is complete rather than batched at the end of the study. Following completion of each PET scan at the study site, the scan data will be promptly sent to the imaging core laboratory (Invicro, LLC) using a cloud-based submission portal. The portal includes automatic pseudonymization of the scan. Full details are in the Study Imaging Manual (also known as Technical Operations Manual).

In addition, results of the image interpretation by the local site reader should be recorded in the eCRF (see Sections 10.2 and 10.3).

11.5 Image Analysis

For the primary endpoint analysis, VOIs will be placed over the urinary bladder and radioactivity (SUV metrics) will be assessed following administration of each radiopharmaceutical.

For secondary and tertiary endpoints, both the piflufolastat (^{18}F) and flotufolastat (^{18}F) PET scans will be read by two independent nuclear medicine physicians or nuclear radiologists who are blinded to the patient's clinical history but have been trained to read scans for each respective radiopharmaceutical using product-specific training and in line with the respective FDA-approved USPI for the respective radiopharmaceutical. To facilitate use of the appropriate product-specific training, the independent readers will be unblinded to the administered agent. Any image interpretation disagreements will be resolved by a third independent adjudicator. Full details are in the Study Image Review Charter.

12 ADVERSE EVENT REPORTING AND DOCUMENTATION

12.1 Adverse Events

An AE is any untoward medical occurrence in a clinical investigation of a patient administered a pharmaceutical product and that does not necessarily have a causal relationship with the product. An AE is therefore any unfavorable and unintended sign (including an abnormal laboratory finding, if applicable), symptom or disease temporally associated with the administration of a pharmaceutical product, whether or not related to that product. An unexpected AE is one of a type not identified in nature, severity, or frequency in the USPI, or of greater severity or frequency than expected based on the information in the USPI.

The collection period for all AEs will begin after informed consent is obtained and end after procedures for the final study visit have been completed (i.e., post-procedural safety monitoring telephone call 24 hours after the flotufolastat (^{18}F) PET scan [Visit 4]). AEs will be assigned as treatment-emergent or non-treatment-emergent as outlined in Table 2.

Table 2: Treatment-emergent Adverse Event Definitions

Time Period	Categorization
• AEs occurring after informed consent (Visit 1) but prior to administration of piflufolastat (^{18}F) (Scan 1; Visit 2)	• Non-treatment-emergent
• AEs occurring from the time of administration of piflufolastat (^{18}F) (Scan 1; Visit 2) to the time of administration of flotufolastat (^{18}F) (Scan 2; Visit 3)	• Treatment-emergent: attributable to piflufolastat (^{18}F)<br>**Note:** any TEAEs attributable to piflufolastat (^{18}F) will not be assigned causality (related/not related to piflufolastat (^{18}F))
• AEs occurring from the time of administration of flotufolastat (^{18}F) (Scan 2; Visit 3) to the final post-flotufolastat (^{18}F) PET safety monitoring telephone call (Visit 4)	• Treatment-emergent: attributable to flotufolastat (^{18}F)

Abbreviations: ^{18}F=fluorine-18; AE=adverse event; PET=positron emission tomography.

As the patient will receive a (^{18}F) PET scan irrespective of their participation in this study, AEs will be collected when the patient returns to the site for Scan 2 (Visit 3). No post-piflufolastat (^{18}F) PET safety monitoring telephone call is planned and AEs will not be solicited, but any AEs volunteered by the patient will be reported in the site's source documents and patient's eCRF. **Suspected adverse reactions for piflufolastat (^{18}F) should be reported to the Marketing Authorization Holder (Progenics Pharmaceuticals, Inc) or the FDA as detailed in the PYLARIFY USPI**.

For the IP, flotufolastat (^{18}F), the investigator (or designee) will probe, via discussion with the patient at the post-procedural safety monitoring telephone call 24 hours after the flotufolastat (^{18}F) PET scan (Visit 4), for the occurrence of AEs and record the information in the site's source documents and patient's eCRF.

Adverse events will be described by duration (start and stop dates and times), severity, outcome, and treatment, and for the IP (flotufolastat (^{18}F)), relationship to IP, or if unrelated, the cause.

12.2 Adverse Event Severity

The National Cancer Institute's Common Terminology Criteria for Adverse Events (CTCAE) Version 5 (November 27, 2017) will be used to assess and grade event severity, including, if applicable, laboratory abnormalities judged to be clinically significant. The severity grading is provided below (Table 3).

Table 3: CTCAE (V5) AE Severity Grading

Severity (Toxicity Grade)	Description
Grade 1	Mild; asymptomatic or mild symptoms; clinical or diagnostic observations only; intervention not indicated.
Grade 2	Moderate; minimal, local, or non-invasive intervention indicated; limiting age-appropriate instrumental ADL.*
Grade 3	Severe or medically significant but not immediately life-threatening; hospitalization or prolongation of hospitalization indicated; disabling; limiting self-care ADL**.
Grade 4	Life-threatening consequences; urgent intervention indicated.
Grade 5	Death related to AE.

Abbreviations: ADL=activities of daily living; AE=adverse event; CTCAE=Common Terminology Criteria for Adverse Events.
*Instrumental ADL refers to preparing meals, shopping for groceries or clothes, using the telephone, managing money, etc.
** Self-care ADL refers to bathing, dressing and undressing, feeding self, using the toilet, taking medications, and not bedridden.

If the experience is not covered in the modified criteria, the guidelines shown in Table 4 below will be used to grade severity. It should be pointed out that the term "severe" is a measure of intensity and that a severe event is not necessarily serious.

Table 4: AE Severity Grading

Severity (Toxicity Grade)	Description
Mild (1)	Transient or mild discomfort; no limitation in activity; no medical intervention or therapy required. The patient may be aware of the sign or symptom but tolerates it reasonably well.
Moderate (2)	Mild to moderate limitation in activity, no or minimal medical intervention/therapy required.
Severe (3)	Marked limitation in activity, medical intervention/therapy required, hospitalizations possible.
Life-threatening (4)	The patient is at risk of death due to the adverse experience as it occurred. This does not refer to an experience that hypothetically might have caused death if it were more severe.
Death (5)	Death related to AE.

Abbreviations: AE=adverse event.

12.3 Adverse Event Relationship to Investigational Product

Any TEAEs attributable to piflufolastat (^{18}F) **will not be** assigned causality (related/not related to piflufolastat (^{18}F)).

The relationship of an AE to the IP (flotufolastat (^{18}F)) will be assessed using the following guidelines in Table 5.

Table 5: AE Relationship to IP (Flotufolastat (^{18}F))

Relationship to IP	Comment
Definitely	Previously known toxicity of agent; or an event that follows a reasonable temporal sequence from administration of the IP; that follows a known or expected response pattern to the suspected IP; that is confirmed by stopping or reducing the dosage of the IP; and that is not explained by any other reasonable hypothesis.
Probably	An event that follows a reasonable temporal sequence from administration of the IP; that follows a known or expected response pattern to the suspected IP; that is confirmed by stopping or reducing the dosage of the IP; and that is unlikely to be explained by the known characteristics of the patient's clinical state or by other interventions.
Possibly	An event that follows a reasonable temporal sequence from administration of the IP; that follows a known or expected response pattern to that suspected IP; but that could readily have been produced by a number of other factors.
Unrelated	An event that can be determined with certainty to have no relationship to the IP.

Abbreviations: ^{18}F=fluorine-18; AE=adverse event; IP=investigational product.

12.4 Serious Adverse Events

A serious adverse event (SAE) is defined as any AE occurring at any dose that results in any of the following outcomes:

- Death;
- A life-threatening adverse experience;
- Inpatient hospitalization or prolongation of existing hospitalization;
- A persistent or significant disability/incapacity;
- A congenital anomaly/birth defect.

Other important medical events may also be considered an SAE when, based on appropriate medical judgment, they jeopardize the patient or require intervention to prevent one of the outcomes listed above.

12.4.1 Serious Adverse Event Reporting

Study sites will document all SAEs reported for piflufolastat (^{18}F) or flotufolastat (^{18}F) in the site's source documents and patient's eCRF. The collection period for SAEs will be the same as that for AEs, as defined in Section 12.1.

All SAEs must be reported using the Electronic Data Capture (EDC) system within 24 hours of the Investigator becoming aware of the event.

The EDC system must be used to complete the SAE report form accurately and comprehensively.

In the event that the EDC system is unavailable, SAEs must be reported via email within the same 24-hour time-frame.

The email must include a completed SAE report form and be sent to the designated safety contact email address: **BED-PSMA-411_SAE@blueearthdx.com.**

Once the EDC system is available, the SAE must be entered into the EDC system as soon as possible, and a note should be made indicating that the initial report was submitted via email.

Additional and further requested information (follow-up or corrections to the original event) will be detailed in EDC or on a new SAE Report Form and emailed to the same address if the EDC is unavailable.

As detailed in Section 12.1, suspected adverse reactions for piflufolastat (^{18}F) should be reported to the Marketing Authorization Holder (Progenics Pharmaceuticals, Inc) or the FDA as detailed in the PYLARIFY USPI.

For the US, in accordance with the standard operating procedures and policies of the local Institutional Review Board (IRB)/Independent Ethics Committee (IEC), the site investigator will report all SAEs to the IRB/IEC.

12.4.2 Suspected Unexpected Serious Adverse Reactions

Suspected unexpected serious adverse reactions (SUSARs) will be collected and reported for the IP, flotufolastat (^{18}F).

A serious adverse reaction is an adverse reaction, the nature and severity of which is not consistent with the reference safety information set out in the approved USPI for flotufolastat (^{18}F).

The sponsor will be responsible for reporting all SUSARs for flotufolastat (^{18}F) to the relevant authorities, IEC/IRB and other parties, as applicable.

Investigators will be informed of all SUSARs for flotufolastat (^{18}F) for all studies sponsored by BED, whether or not the event occurred in the current study.

As detailed in Section 12.1, suspected adverse reactions for piflufolastat (^{18}F) should be reported to the Marketing Authorization Holder (Progenics Pharmaceuticals, Inc) or the FDA as detailed in the PYLARIFY USPI.

12.5 Medical Monitoring

Phillip B. Davis, MD should be contacted directly at this numbers to report medical concerns or questions regarding safety:

Telephone: +1 843 367 4652

13 DISCONTINUATION AND REPLACEMENT OF PATIENTS

13.1 Discontinuation/Withdrawal From the Study

Patients are free to withdraw from participation in the study at any time, for any reason, specified or unspecified, and without prejudice.

An investigator may discontinue or withdraw a patient for the following reasons:

- Significant non-compliance;
- If any clinical AE, laboratory abnormality, or other medical condition or situation occurs such that continued participation in the study would not be in the best interests of the patient;
- Protocol violation requiring discontinuation of study treatment;
- If the patient meets any exclusion criteria (either newly developed or not previously recognized) that precludes further study participation;
- Lost to follow-up;
- Sponsor request for early termination of study.

The reason for patient discontinuation or withdrawal from the study, if known, will be recorded in the patient's source documents and in the eCRF.

13.2 Additional Patients

In order to obtain a minimum of 52 evaluable patients (i.e., have evaluable PET scans for both piflufolastat (^{18}F) and flotufolastat (^{18}F) available for analysis), additional patients may be recruited if any patient does not receive **both** radiopharmaceuticals and have an evaluable PET scan for each.

13.3 Lost to Follow-up

A patient will be considered lost to follow-up if they fail to return for the second PET scan (flotufolastat (^{18}F)) within 10 calendar days of the first PET scan (piflufolastat (^{18}F)) and is unable to be contacted by the study site staff.

The following actions must be taken if a patient fails to return to the clinic for a required study visit:

- The site will attempt to contact the patient and reschedule the missed visit (no later than within 10 calendar days of the first PET scan) and counsel the patient on the importance of maintaining the assigned visit schedule and ascertain if the patient wishes to and/or should continue in the study;
- Before a patient is deemed lost to follow-up, the investigator or designee will make every effort to regain contact with the patient (where possible, three attempts to contact). These contact attempts should be documented in the patient's medical record or study file;
- Should the patient continue to be unreachable, they will be considered to have withdrawn from the study with a primary reason of lost to follow-up.

14 PROTOCOL VIOLATIONS

A protocol violation occurs when the patient, investigator, or sponsor fails to adhere to significant protocol requirements affecting the inclusion, exclusion, patient safety, and primary endpoint criteria. Protocol violations for this study include, but are not limited to, the following:

- Failure to meet inclusion/exclusion criteria (see Sections 6.2 and 6.3);
- Use of a prohibited concomitant medication (see Section 7.2);
- Radiopharmaceutical administration but no subsequent PET scan;
- Use of a non-approved PET/CT scanner.

Significant non-compliance with Good Clinical Practice (GCP) guidelines will also result in a protocol violation. The sponsor will determine if a protocol violation will result in the withdrawal of a patient.

When a protocol violation occurs, it will be discussed with the investigator and a protocol violation form detailing the violation will be generated. This form will be signed by a sponsor representative and the investigator. A copy of the form will be filed in the investigator site's file and in the sponsor's files.

15 STATISTICAL METHODS AND CONSIDERATIONS

Prior to the analysis of the final study data, a detailed Statistical Analysis Plan (SAP) will be written describing all analyses that will be performed. The SAP will contain any modifications to the analysis plan described below.

15.1 Estimation of Sample Size

Approximately 60 patients will be screened for inclusion into the study to obtain a minimum of 52 evaluable patients (i.e., have evaluable PET scans for both piflufolastat (^{18}F) and flotufolastat (^{18}F) available for analysis).

A sample size of 52 evaluable patients achieves 80% power to detect a mean of paired differences of 10 in SUV_{mean}, with an estimated standard deviation of paired differences of 25 and with a significance level of 0.05 using a two-sided paired Wilcoxon signed-rank test assuming that the actual distribution of paired differences is uniform.

The difference in SUV_{mean} of 10 is based on the difference in the median bladder SUV_{mean} values of 25.4 observed for another renally cleared PSMA PET ligand, ^{68}Ga-PSMA-11 (Kuten et al, 2020) and 12.5 observed for flotufolastat (^{18}F) (Kuo et al, 2024; see Figure 3, Section 1.3).

15.2 Data Sets to be Analyzed

All Enrolled Patients: All patients who sign the Informed Consent Form (ICF).

Full Analysis Set (FAS): All patients who receive either piflufolastat (^{18}F) or flotufolastat (^{18}F), and the corresponding PET scan is evaluable.

Full Safety Set (SAF): All patients who receive either piflufolastat (^{18}F) or flotufolastat (^{18}F), regardless of whether the corresponding PET scan is performed.

Efficacy Analysis Set (EAS): All patients who receive both piflufolastat (^{18}F) and flotufolastat (^{18}F), and the corresponding PET scans are evaluable.

Per Protocol Set (PPS): All patients who receive both piflufolastat (^{18}F) and flotufolastat (^{18}F), and the corresponding PET scans are evaluable, and who are without any major protocol deviations that will affect the evaluations of the PET scans.

15.3 Demographic and Baseline Characteristics

The following demographic variables at screening (Visit 1) will be summarized: age, ethnicity/race, and height. Weight and body mass index at screening (Visit 1) and on the day of each PET scan (Visits 2 and 3) will be summarized.

Medical history, including general medical history and prostate cancer history (diagnosis and prior therapies) will be summarized. In addition, PSA values collected at screening (within 14 days of Visit 2) will be summarized.

15.4 Analysis of Primary Endpoint(s)

The primary endpoint will be the difference in urinary bladder SUV_{mean} between piflufolastat (^{18}F) and flotufolastat (^{18}F). This will be calculated for each patient. A two-sided paired Wilcoxon Signed Rank Test will be used to test the difference in urinary bladder SUV_{mean} between piflufolastat (^{18}F) and flotufolastat (^{18}F). This will be performed in patients who have received both radiopharmaceuticals and have both PET scans available.

15.5 Analysis of Secondary and Tertiary Endpoint(s)

Secondary and tertiary endpoints will be summarized descriptively, with 95% confidence intervals presented, where applicable. Further details will be provided in the SAP.

Safety data will be summarized descriptively for the IP.

All AEs will be coded using the Medical Dictionary for Regulatory Activities (MedDRA) and TEAEs will be summarized overall and by severity for piflufolastat (^{18}F) and flotufolastat (^{18}F) and will include the number and percentage of patients for whom each event occurred. For flotufolastat (^{18}F) only, TEAEs will also be summarized by relationship to IP.

15.6 Planned Interim Analysis

Not applicable.

16 DATA COLLECTION, RETENTION AND MONITORING

16.1 Data Collection Instruments

The investigator will prepare and maintain adequate and accurate source documents designed to record all observations and other pertinent data for each patient treated with the IP. Particular care should be taken to ensure all data points are recorded in source documentation, especially those which are not part of standard practice.

Study personnel at each site will enter data from source documents corresponding to a patient's visit into the protocol-specific eCRF when the information corresponding to that visit is available.

Patients will not be identified by name in the study database or on any study documents to be collected by the sponsor (or designee) but will be identified by a site number and patient number. Only partial date of birth (i.e., year) will be collected.

If a correction is required for an eCRF, the time and date stamps track the person entering or updating eCRF data and creates an electronic audit trail.

The investigator is responsible for all information collected on patients enrolled in this study. All data collected during the course of this study must be reviewed and verified for completeness and accuracy by the investigator. A copy of the eCRF will remain at the investigator's site at the completion of the study.

16.2 Data Management Procedures

Clinical data will be entered by site into a validated database, and the independent evaluation of the images will be entered into another validated database by the PET readers. The respective Data Management groups will be responsible for data processing, in accordance with procedural documentation. Database lock will occur once quality assurance procedures have been completed.

All procedures for the handling and analysis of data will be conducted in accordance with applicable data standards and data cleaning procedures to ensure the integrity of the data meets FDA guidelines for the handling and analysis of data for clinical trials.

16.3 Data Quality Control and Reporting

After data has been entered into the study database, a system of computerized data validation checks will be implemented and applied to the database on a regular basis. Queries will be entered, tracked, and resolved through the Electronic Data Capture (EDC) system directly. The study database will be updated in accordance with the resolved queries. All changes to the study database will be documented.

Following completion of each PET scan at the study site, the scan data will be promptly sent to the imaging core laboratory (Invicro, LLC) rather than batched at the end of the study (see Section 11.4). Image data submitted to the imaging core laboratory (Invicro, LLC) will be quality assessed to verify that the metadata are consistent with study patient information, and that all images are evaluable, before entering image-associated information into the image tracking software application. Queries will be entered, tracked, and resolved through the image tracking software application directly.

16.4 Archiving of Data

The database is safeguarded against unauthorized access by established security procedures; appropriate backup copies of the database and related software files will be maintained. Databases are backed up by the database administrator in conjunction with any updates or changes to the database.

At critical junctures of the protocol (e.g., production of final reports), data for analysis is locked and cleaned per established procedures.

16.5 Availability and Retention of Investigational Records

The investigator/institution should maintain adequate and accurate source documents and study records that include all pertinent observations on each of the site's study patients. Source data should be attributable, legible, contemporaneous, original, accurate and complete. Changes to source data should be traceable, should not obscure the original entry, and should be explained if necessary.

The investigator/institution should maintain the study documents as specified in Section 8 of International Council for Harmonisation (ICH) GCP E6 (R2) and as required by the applicable regulatory requirement(s). The investigator/institution should take measures to prevent accidental or premature destruction of these documents.

Upon request of the monitor, auditor, IRB/IEC, or regulatory authority, the investigator/institution must make available for direct access all requested study-related records.

Study records should be retained for at least 25 years after the end of the clinical trial. These documents should be retained for a longer period, however, if required by applicable local regulations. No records will be destroyed without the written consent of the sponsor, if applicable. It is the responsibility of the sponsor to inform the investigator when these documents no longer need to be retained.

The medical files of patients shall be archived in accordance with applicable local regulations.

16.6 Monitoring and Auditing

Monitoring visits will be conducted by representatives of the sponsor according to ICH GCP and relevant regulations. By signing this protocol, the investigator grants permission to the sponsor's (or designee's) monitors and auditors, as well as the IRB/IEC and regulatory authorities to conduct on-site monitoring and/or auditing and provide direct access to all requested study-related records.

16.7 Patient Confidentiality

In order to maintain patient confidentiality, only a site number, patient number, and partial date of birth (i.e., year) will identify all study patients in the eCRFs and other documentation submitted to the sponsor. Additional patient confidentiality issues (if applicable) are covered in the Clinical Study Agreement.

To maintain confidentiality, all laboratory specimens, evaluation forms, reports and other records will be identified by a coded number and initials only. All study records will be kept in a locked file cabinet and code sheets linking a patient's name to a patient identification number will be stored separately in another locked file cabinet.

Clinical information will not be released without written permission of the patient, except as necessary for monitoring by regulatory authorities. The investigator must also comply with all

applicable privacy regulations (e.g., HIPAA [1996], EU General Data Protection Regulation [2016/679] etc.]).

17 REGULATORY, ETHICAL AND STUDY OVERSIGHT CONSIDERATIONS

The study will be conducted in accordance with ICH GCP and all applicable regulations. The investigator will assure that no deviation from, or changes to the protocol will take place without prior agreement from the sponsor and documented approval from the relevant regulatory authority (if applicable) and IRB/IEC, except where necessary to eliminate an immediate hazard(s) to the trial patients. All personnel involved in the conduct of this study have completed ICH GCP training, relevant to their role.

17.1 Institutional Review Boards and Independent Ethics Committees

Any documents that the IRB/IEC may need to fulfill its responsibilities (such as the protocol, protocol amendments, IB, ICFs, information concerning patient recruitment, payment or compensation procedures, or other pertinent information) will be submitted to the IRB/IEC.

The IRB/IECs written unconditional approval/favorable opinion of the study, and any additional local approvals (e.g., hospital management, etc.), must be obtained prior to shipment of the IP to the site and prior to any patients undergoing study-specific procedures. The investigator will obtain assurance of IRB/IEC compliance with regulations.

Note: Regulatory authority approvals may also be required.

The IRB/IEC's standard operating procedures and policies will be followed for the submission of SAEs and progress reports during the conduct of the study.

An end of study notification will be submitted as determined by each countries regulatory requirements.

17.2 Amendments

Any decision to amend the clinical trial application and/or associated documents (e.g., protocol, ICF, eCRF, IB etc.) will be made by the sponsor/contract research organization.

The relevant regulations will be followed to determine what approvals from regulatory, IRB/IEC or local bodies are required. All required approvals will be obtained prior to implementation of the amendment, except as necessary to eliminate immediate safety hazards to patients. The sponsor/contract research organization will notify each participating investigator site when the amendment can be implemented.

All changes to the ICF will be IRB/IEC approved; a determination will be made regarding whether a new consent needs to be obtained from patients who provided consent, using a previously approved ICF.

17.3 Patient Information and Consent

In obtaining and documenting patient informed consent, the investigator must comply with the application regulatory requirement(s), ICH GCP and the ethical principles that have their origin in the Declaration of Helsinki.

Patient information and ICFs, and any other written material provided to the patient, must be approved by the relevant IRB/IEC (and by any other body as required by national regulations) prior to the start of the study at each study site.

The investigator (or an appropriately qualified designee) will explain the study to the patient and answer any questions that arise. A verbal explanation will be provided in terms suited to the patient's comprehension of the purposes, procedures and potential risks of the study, and

the rights of research patients. Patients will have the opportunity to carefully review the written information and ICF, to discuss the study with their family or surrogates, and be given ample time to think about the study and ask questions before agreeing to participate.

Patients must be informed that participation is voluntary and that they may withdraw from the study at any time, without prejudice. The rights and welfare of the patients will be protected by emphasizing to them that the quality of their medical care will not be adversely affected if they decline to participate in this study.

Prior to the patient undergoing any study-specific procedures, the written ICF must be signed and personally dated by the patient and by the person who conducted the informed consent discussion. The informed consent process will also be documented in the source document (including the date/time consent was obtained).

If a patient is unable to read, an impartial witness should be present during the entire informed consent discussion. The patient may orally consent to the patient's participation if the patient is not capable of signing and personally dating the ICF. Once the patient has provided consent, the witness should also sign and personally date the ICF. By signing the ICF the witness attests that the information sheet/ICF was accurately explained to, and apparently understood by, the patient and that informed consent was freely given by the patient.

The distribution of the signed information sheet/ICF will be as required by any applicable local regulations. Otherwise, a copy of the signed ICF will be given to the patient and the original maintained with the patient's records.

The patient will be informed in a timely manner if new information becomes available that may be relevant to the patient's willingness to continue participation in the study. The communication of this information will be documented in the source documentation. The written patient information/ICF and any other written information provided to the patients should be revised whenever important new information becomes available that may be relevant to the patient's consent. Any revised written patient information and ICF should receive IRB/IEC approval/favorable opinion prior to use. The patient should sign and personally date any revised ICF and receive a copy (or original, if required by applicable regulations).

17.4 Post-trial Care

Both radiopharmaceuticals used in this Phase 4 study are single-use diagnostic agents. No additional care for trial patients is therefore planned once their participation in the study has ended. All patients will receive standard of care treatment in line with their medical condition as determined by their physician.

17.5 Publications

The preparation and submittal for publication of manuscripts containing the study results shall be in accordance with a process determined by mutual written agreement among the study sponsor and participating institutions. The publication or presentation of any study results shall comply with all applicable privacy laws, including, but not limited to, HIPAA (1996) and the EU General Data Protection Regulation (2016/679).

17.6 Investigator Responsibilities

By signing the Protocol Agreement form, the investigator agrees to:

1. Comply with ICH GCP principles and all applicable regulatory requirements; be familiar with the appropriate use of the radiopharmaceuticals as described in the protocol, IB, respective USPIs and any other information sources provided.

2. Personally conduct or supervise the study; maintain a list of appropriately qualified persons to whom significant trial-related duties are delegated. Ensure all persons assisting with the study are adequately informed about the protocol, the radiopharmaceuticals, and their study-related duties.
3. Ensure that all study-related medical decisions are made by a qualified physician who is an investigator or sub-investigator for the study; ensure that adequate medical care is provided to a patient for any AEs.
4. Obtain the written approval/favorable opinion of the IRB/IEC before the study starts; provide the IRB/IEC with the current IB and all documents subject to review throughout the trial.
5. Following completion of each PET scan at the study site, promptly send the scan data to the imaging core laboratory (Invicro, LLC) using the cloud-based submission portal.
6. Submit a written progress report at least annually and in accordance with the IRB/IEC's request; submit an end of trial notification/final report to the IRB/IEC at the end of the study.
7. Conduct the study in compliance with the approved protocol and not implement any deviation from, or changes to the protocol without the agreement of the sponsor (or designee), and prior approval/favorable opinion of the IRB/IEC of an amendment, except where necessary to eliminate an immediate hazard to a study patient, or when the change(s) involve only logistical or administrative aspects of the trial.
8. Promptly (immediately) notify the sponsor (or designee) and IRB/IEC of any actions taken to eliminate an immediate hazard to a study patient.
9. Comply with ICH GCP, applicable regulatory requirements and the ethical principles that have their origin in the Declaration of Helsinki, when obtaining and documenting informed consent.
10. Ensure that the radiopharmaceuticals are stored as specified by the sponsor per the respective USPIs and in accordance with applicable regulatory requirement(s); used in accordance with the protocol and respective USPIs, and that adequate records are maintained.
11. Immediately report all SAEs to the sponsor unless otherwise specified in the protocol or other document (e.g., IB). Comply with applicable regulatory requirement(s) related to the reporting of unexpected serious adverse drug reactions to regulatory authorities and the IRB/IEC.
12. If the study is prematurely terminated or suspended for any reason, promptly inform study patients, and assure appropriate therapy and follow-up, as required; follow ICH GCP and required regulatory requirements to notify the sponsor and IRB/IEC.
13. Maintain adequate and accurate source documents and study records that include all pertinent observations on each of the study patients; source data should be attributable, legible, contemporaneous, original, accurate and complete. Changes to source data must be traceable, should not obscure the original entry, and should be explained if necessary.
14. Retain essential documents for at least 25 years or until notified by the sponsor.
15. Provide monitors, auditors, IRB/IEC and regulatory authorities direct access to all requested study-related records.

18 REFERENCES

Afshar-Oromieh A, Haberkorn U, Schlemmer HP, et al. Comparison of PET/CT and PET/MRI hybrid systems using a ^{68}Ga-labelled PSMA ligand for the diagnosis of recurrent prostate cancer: initial experience. Eur J Nucl Med Mol Imaging. 2014;41(5):887-97.

Chapin BF. Diagnostic performance and safety of ^{18}F-rhPSMA-7.3 PET in patients with newly diagnosed prostate cancer: results from a phase 3, prospective, multicenter study (LIGHTHOUSE). Society of Urologic Oncology, Inc. 22nd Annual Meeting 2022. Available at: https://suo-abstracts.secure-platform.com/a/gallery/rounds/15/details/2390 [Accessed 08 February 2024].

Donswijk ML, Wondergem M, de Wit-van der Veen L, et al. Effects of furosemide and tracer selection on urinary activity and peri-bladder artefacts in PSMA PET/CT: a single-centre retrospective study. EJNMMI Res. 2022;12(1):42.

Fleming, M. PLLBA-02. Impact of ^{18}F-rhPSMA-7.3 PET on upstaging of patients with prostate cancer recurrence: results from the prospective, phase 3, multicenter, SPOTLIGHT study. J Urol. 2022;207(5S, Suppl):e1047.

Giesel FL, Will L, Lawal I, et al. Intraindividual comparison of ^{18}F-PSMA-1007 and ^{18}F-DCFPyL PET/CT in the prospective evaluation of patients with newly diagnosed prostate carcinoma: a pilot study. J Nucl Med. 2018;59(7):1076-80.

Helfand BT. MP40-13. ^{18}F-rhPSMA-7.3 detection rates in patients with recurrence of prostate cancer following primary treatment with radiation therapy: results from the SPOTLIGHT study. J Urol. 2023;209(4S, Suppl):e549-50.

Heußer T, Mann P, Rank CM, et al. Investigation of the halo-artifact in ^{68}GaPSMA11PET/MRI. PLoS One. 2017;12(8):e0183329.

Jani AB, Ravizzini GC, Gartrell BA, et al. Diagnostic performance and safety of ^{18}F-rhPSMA 7.3 positron emission tomography in men with suspected prostate cancer recurrence: results from a phase 3, prospective, multicenter study (SPOTLIGHT). J Urol. 2023;210(2):299-311.

Knorr K, Oh SW, Krönke M, et al. Preclinical biodistribution and dosimetry and human biodistribution comparing ^{18}F-rhPSMA-7 and single isomer ^{18}F-rhPSMA-7.3. EJNMMI Res. 2022;12(1):8.

Kroenke M, Schweiger L, Horn T, et al. Validation of ^{18}F-rhPSMA-7 and ^{18}F-rhPSMA-7.3 PET imaging results with histopathology from salvage surgery in patients with biochemical recurrence of prostate cancer. J Nucl Med. 2022;63(12):1809-14.

Kuo P, Esposito G, Yoo D, et al. Inter- and intra-reader reproducibility of ^{18}F-rhPSMA-7.3 PET image interpretation in patients with suspected prostate cancer recurrence: results from a phase 3, prospective, multicenter study (SPOTLIGHT). J Nucl Med. 2022;63(2, Suppl):2539.

Kuo PH, Hermsen R, Penny R, et al. Quantitative and qualitative assessment of urinary activity of ^{18}F-flotufolastat-PET/CT in patients with prostate cancer: a post hoc analysis of the LIGHTHOUSE and SPOTLIGHT studies. Mol Imaging Biol. 2024;26(1):53-6.

Kuo P, Ravizzini G, Ulaner G, et al. Inter- and intra-reader reproducibility of ^{18}F rhPSMA-7.3 PET interpretation in patients with newly diagnosed prostate cancer: results from a phase 3, prospective, multicenter study (LIGHTHOUSE). J Nucl Med. 2023;64(1, Suppl):P589.

Kuten J, Fahoum I, Savin Z, et al. Head-to-head comparison of ^{68}Ga-PSMA-11 with ^{18}F-PSMA-1007 PET/CT in staging prostate cancer using histopathology and immunohistochemical analysis as a reference standard. J Nucl Med. 2020;61(4):527-32.

Langbein T, Wang H, Rauscher I, et al. Utility of ^{18}F-rhPSMA-7.3 PET for imaging of primary prostate cancer and pre-operative efficacy in N-staging of unfavorable intermediate- to very high-risk patients validated by histopathology. J Nucl Med. 2022;63(9):1334-42.

Lowentritt B. Impact of clinical factors on ^{18}F-rhPSMA-7.3 detection rates in men with recurrent prostate cancer: findings from the phase 3 SPOTLIGHT study. Int J Radiat Oncol Biol Phys. 2022;114(3, Suppl):S130-1.

Malaspina S, Oikonen V, Kuisma A, et al. Kinetic analysis and optimisation of ^{18}FrhPSMA7.3 PET imaging of prostate cancer. Eur J Nucl Med Mol Imaging. 2021;48(11):3723-31.

Malaspina S, Taimen P, Kallajoki M, et al. Uptake of ^{18}F-rhPSMA-7.3 in positron emission tomography imaging of prostate cancer: a phase 1 proof-of-concept study. Cancer Biother Radiopharm. 2022;37(3):205-13.

PYLARIFY® [piflufolastat F 18] injection USPI. Progenics Pharmaceuticals, Inc. 2021. Available at: https://www.accessdata.fda.gov/drugsatfda_docs/label/2021/214793s000lbl.pdf [Accessed 08 February 2024]

POSLUMA® [flotufolastat F 18] injection USPI. Blue Earth Diagnostics 2023. Available at: https://www.accessdata.fda.gov/drugsatfda_docs/label/2023/216023s000lbl.pdf [Accessed 08 February 2024]

Rauscher I, Karimzadeh A, Schiller K, et al. Detection efficacy of ^{18}F-rhPSMA-7.3 PET/CT and impact on patient management in patients with biochemical recurrence of prostate cancer after radical prostatectomy and prior to potential salvage treatment. J Nucl Med. 2021;62(12):1719-26.

Rauscher I, Maurer T, Fendler WP, et al. (68)Ga-PSMA ligand PET/CT in patients with prostate cancer: how we review and report. Cancer Imaging. 2016;16(1):14.

Surasi DS, Eiber M, Maurer T, et al. Diagnostic performance and safety of positron emission tomography with ^{18}F-rhPSMA-7.3 in patients with newly diagnosed unfavourable intermediate- to very-high-risk prostate cancer: results from a phase 3, prospective, multicentre study (LIGHTHOUSE). Eur Urol. 2023;84(4):361-70.

Tolvanen T, Kalliokoski K, Malaspina S, et al. Safety, biodistribution, and radiation dosimetry of ^{18}F-rhPSMA-7.3 in healthy adult volunteers. J Nucl Med. 2021;62(5):679-84.

Uprimny C, Bayerschmidt S, Kroiss AS, et al. Impact of forced diuresis with furosemide and hydration on the halo artefact and intensity of tracer accumulation in the urinary bladder and kidneys on [^{68}Ga]Ga-PSMA-11-PET/CT in the evaluation of prostate cancer patients. Eur J Nucl Med Mol Imaging. 2021;48(1):123-33.

Wondergem M, van der Zant FM, Rafimanesh-Sadr L, et al. Effect of forced diuresis during ^{18}F-DCFPyL PET/CT in patients with prostate cancer: activity in ureters, kidneys and bladder and occurrence of halo artefacts around kidneys and bladder. Nucl Med Commun. 2019;40(6):652-6.

Wurzer A, Di Carlo D, Schmidt A, et al. Radiohybrid ligands: a novel tracer concept exemplified by ^{18}F- or ^{68}Ga-labeled rhPSMA inhibitors. J Nucl Med. 2020;61(5):735-42.

APPENDIX 1. PROTOCOL AGREEMENT

I have read the protocol specified below. In my formal capacity as investigator, my duties include providing Blue Earth Diagnostics with the information, as outlined in the protocol. It is understood that all information pertaining to the study will be held strictly confidential and that this confidentiality requirement applies to all study staff at this site. Furthermore, on behalf of the study staff and myself, I agree to maintain the procedures required to carry out the study in accordance with accepted Good Clinical Practice principles, applicable regulatory requirements, and to abide by the terms of this protocol.

Protocol Number: BED-PSMA-411

Protocol Title: Intra-patient Comparison of Urinary Radioactivity Following Piflufolastat (^{18}F) and Flotufolastat (^{18}F) PET in Men with Low PSA Biochemical Recurrence of Prostate Cancer Following Radical Prostatectomy

Protocol Version: 4.0 Protocol Date: 22-Apr-2025

____ __________________

Investigator Signature Date

____

Print Name and Title

Site # __________

Site Name ____

Address ____

____

____

____

Phone Number ____
